# Putative SF2 helicases of the early-branching eukaryote *Giardia lamblia* are involved in antigenic variation and parasite differentiation into cysts

**DOI:** 10.1186/1471-2180-12-284

**Published:** 2012-11-28

**Authors:** Pablo R Gargantini, Marianela C Serradell, Alessandro Torri, Hugo D Lujan

**Affiliations:** 1 Laboratory of Biochemistry and Molecular Biology, School of Medicine, Catholic University of Córdoba, Córdoba, X5004ASK, Argentina

**Keywords:** RNA/DNA helicases, Giardia lamblia, Encystation, Antigenic variation, Cell differentiation, Gene expression, RNAi, Dicer

## Abstract

**Background:**

Regulation of surface antigenic variation in *Giardia lamblia* is controlled post-transcriptionally by an RNA-interference (RNAi) pathway that includes a Dicer-like bidentate RNase III (gDicer). This enzyme, however, lacks the RNA helicase domain present in Dicer enzymes from higher eukaryotes. The participation of several RNA helicases in practically all organisms in which RNAi was studied suggests that RNA helicases are potentially involved in antigenic variation, as well as during *Giardia* differentiation into cysts.

**Results:**

An extensive *in silico* analysis of the *Giardia* genome identified 32 putative Super Family 2 RNA helicases that contain almost all the conserved RNA helicase motifs. Phylogenetic studies and sequence analysis separated them into 22 DEAD-box, 6 DEAH-box and 4 Ski2p-box RNA helicases, some of which are homologs of well-characterized helicases from higher organisms. No *Giardia* putative helicase was found to have significant homology to the RNA helicase domain of Dicer enzymes. Additionally a series of up- and down-regulated putative RNA helicases were found during encystation and antigenic variation by qPCR experiments. Finally, we were able to recognize 14 additional putative helicases from three different families (RecQ family, Swi2/Snf2 and Rad3 family) that could be considered DNA helicases.

**Conclusions:**

This is the first comprehensive analysis of the Super Family 2 helicases from the human intestinal parasite *G. lamblia*. The relative and variable expression of particular RNA helicases during both antigenic variation and encystation agrees with the proposed participation of these enzymes during both adaptive processes. The putatives RNA and DNA helicases identified in this early-branching eukaryote provide initial information regarding the biological role of these enzymes in cell adaptation and differentiation.

## Background

Helicases are encoded by a large fraction of prokaryotic and eukaryotic genomes and are found in all organisms –from bacteria to humans– and in many viruses. These nucleic acid-dependent NTPases (preferentially ATPases) have the ability to unwind DNA or RNA duplex substrates; to unwind/separate the helical structure of double-stranded nucleic acids and, in some cases, to disrupt protein-nucleic acid interactions [1,2].

DNA and RNA helicases are grouped into six superfamilies (SF). SF1 and SF2 do not form rings, whereas SF3 to SF6 comprise the ring-forming helicases [3]. All eukaryotic RNA helicases belong to SF1 and SF2, whereas the ring-shaped RNA helicases are found in viruses [4] and bacteria [5,6]. Functional groups for ATP binding and hydrolysis are highly conserved among SF1 and SF2 DNA and RNA helicases. In addition, these two superfamilies show high sequence similarity in their conserved regions, sharing eight conserved motifs; and variations within these conserved motifs are used to distinguish between these very closely related families.

The helicases from SF1 and SF2 are further divided into families, based on their sequence, structural, and mechanistic features [3,7]. According to an excellent classification proposed by Jankowsky’s group, these helicases can be grouped into three families in the SF1 and nine families and one group in the SF2 [8]. Although several helicase families contain both RNA and DNA helicases, six of these twelve families only contain RNA helicases (DEAD-box, DEAH-box, Ski2-like, RIG-I-like, NS3/NPH-II and Upf1-like families). As they are mainly composed by RNA helicases, these 6 families are termed “RNA helicase families”, and are often referred to as DExD/H proteins.

In the SF1 and SF2 helicases, the conserved motifs are clustered in a “central” core region that spans about 350 to 400 amino acids (named “Helicase Core Domain” - HCD). By contrast, the N- and C-terminal extensions of helicases are highly variable in size and composition. These regions are supposed to confer substrate specificity, comprising protein- and/or RNA-binding motifs that provide helicases with their capacity to be involved in multiple processes, and/or direct the helicases to their subcellular localization [9,10]. Within these extensions helicases also contain accessory domains that can confer specific functions, as in the case of the bidentate RNase III enzyme Dicer [11]. The conservation of these domains within a family is null; therefore, they are not used to define a typical group.

RNA is involved in virtually all aspects of gene expression, playing important regulatory roles in biological reactions and making RNAs biologically important molecules required by all living organisms. RNA helicases may act as a temporary clamp to prevent RNAs from re-associating, thereby allowing other RNA-RNA or RNA-protein interactions to occur. A recent report suggests that these proteins can also function as RNPases [12], which are enzymes that disrupt RNA-protein interactions.

*Giardia lamblia* is a single-celled eukaryotic microorganism that inhabits in the upper small intestine of humans and several other vertebrates. Phylogenetic studies have placed *Giardia* as one of the most early-branching eukaryotic cells [13-17]. In addition to its biological relevance, *G. lamblia* is one of the leading causes of human intestinal disease worldwide, the most frequent cause of defined waterborne outbreaks of diarrhea in developed countries and a common cause of diarrhea in daycare centers, institutionalized individuals, backpackers, and travelers [18]. The parasite has a simple life cycle, comprising the disease-causing trophozoites and the environmentally-resistant cysts, which are responsible for the transmission of the disease among susceptible hosts [18]. *Giardia* undergoes important adaptive mechanisms to survive both inside and outside the host’s intestine, such as “antigenic variation” and “encystation”, respectively [19]. Antigenic variation is characterized by the continuous switching of surface antigenic molecules, which allows the parasite to evade the immune response generated by the host [20]. In *Giardia*, antigenic variation involves variant-specific surface proteins (VSPs), cysteine-rich type 1a membrane proteins that cover the entire surface of the trophozoites [21]. Only one VSP, of approximately 200 VSP genes present in the parasite’s genome, is expressed on the surface of individual trophozoites at a given time, but switching to a different VSP occurs once every 6-18 generations. Antigenic variation in *Giardia* is regulated post-transcriptionally by a mechanism similar to RNA interference (RNAi) [22]. Notably, disruption of the RNAi pathway by knocking-down the expression of the dsRNA endonuclease Dicer promotes a change from single to multiple VSP expression on the surface of individual *Giardia* cells, indicating the direct involvement of this enzyme in controlling antigenic variation in this parasite [23]. Nonetheless, gDicer lacks the C-terminal RNA helicase domain, raising question about the function of this domain in Dicer enzymes of higher eukaryotes.

*G. lamblia* possesses functional RNAi machinery [22]. However, this early-branching eukaryote lacks Drosha and Exportin 5 molecules needed to process and export miRNA from the cell nucleus into the cytoplasm as well as other essential components of the RNAi machinery found in higher eukaryotes [24]. It was recently proposed, however, that lack of Drosha and Exportin 5 in *Giardia* could be bypassed by the use of snoRNAs as miRNAs precursors [25]. Interestingly, *Giardia* Dicer is still capable of robust dicing and of complementing the lack of a functional Dicer in *Schizosaccharomyces pombe* (which possesses the RNA helicase domain [26]) as well as ORF-derived miRNAs with gDicer apparently assuming the functions of both Drosha and Dicer [25]. Hence, two questions arise: (i) Are RNA helicases truly involved in the *Giardia* RNAi pathway? (ii) What is the minimal protein repertoire for post-transcriptional gene silencing in eukaryotic cells?

In the present study, we identified the complete set of SF2 helicases in this anaerobic flagellated protozoan by searching the *G. lamblia* genome database of the WB isolate, which allowed the identification of 22 DEAD-box, 6 DEAH-box and 4 Ski2p putative RNA helicases, along with seven helicases of family Swi2/Snf2, 3 helicases from family RecQ and 4 helicases from family Rad3. These sequences were used to analyze the relationship between the composition of the SF2 helicases in *Giardia* and their corresponding homologs in yeast and humans. In addition, the level of expression during antigenic variation and encystation was analyzed, demonstrating both differential and variable expression of individual RNA helicases in these processes. We also discuss the potential role of the RNA helicase domain in Dicer enzymes of higher eukaryotes.

## Results

### Identification of SF2 helicases in *Giardia lamblia*

By using the human eIF4A (Eukaryotic Initiation Factor 4A) amino acid sequence as the DEAD-box helicase prototype [27] and the human ATP-dependent RNA-helicase DHX8 amino acid sequence as the DEAH-box helicase prototype [27], we performed an extensive analysis of the *Giardia* assemblage A, isolate WB, genome database [28] and detected 22 and 6 orthologs, respectively. We were also able to obtain the sequences of 4 putative RNA helicases belonging to the Ski2 family, which is generally classified inside the DExH-box family; and a previously described UPF1 homolog from SF1 [29]. These helicases belong to three of the nine families described from SF2. Therefore, in an attempt to identify any other helicase from this superfamily we performed a PSI-BLASTP search within the *Giardia* genome using the sequences described from humans, yeast and *Escherichia coli*, following Fairman-Williams [8]. Using this approach, we were able to recognize 14 additional putative helicases from three different families, 3 helicases from the RecQ family, 7 helicases from the Swi2/Snf2 family, and 4 helicases from the Rad3 family. The sequences from the remaining three families of SF2 helicases present in humans, yeast and *E. coli* (RecG-like, RIG-I-like and NS3/NPH-II) do not have significant homology with any gene of *G. lamblia*.

The *Giardia* Database gene number, the Contig number and position, and the gene length and codified protein molecular weight for each one of the SF2 helicases studied in this work are summarized in Additional file 1: Table S1. The HCD is virtually conserved in length between the three RNA helicases families, ranging from 361 to 425 amino acids, whereas the greatest differences found, as expected, were in the N- and C-terminal regions of each helicase family (see Additional file 2: Table S2).

The presence of 8 to 10 motifs considered as “signature sequences” were useful for the classification of each putative RNA helicase within a specific family. This approach has already been used to identify DExD/H helicases in human, yeast, rice, *Entamoeba histolytica*, *Plasmodium falciparum*, *Leishmania major*, *Trypanosoma cruzi* and *Trypanosoma brucei* (Table 1). The relationship between the number of DEAD-box and DExH-box helicases supports our finding of 22 DEAD-box and 10 DExH-box (6 DEAH-box and 4 Ski2-like) in *Giardia*. Multiple sequence analysis generated a phylogenetic tree, showing the evolutionary separation of these six families (DEAD-box, DEAH-box, Ski2, RecQ, Rad3, and Swi2/Snf2) (see Additional file 3: Figure S1).

**Table 1:** Number of putative DExD/H-box RNA helicases in other organisms

**Organism**	**DExD/H helicase family**		**(Reference)**
	**DEAD-box**	DExH-box*	
*Giardia lamblia*	**22**	**10**	
*Homo sapiens*	**42**	**18**	[30]
*Oryza sativa*	**26**	**8**	[31]
*Saccharomyces cerevisiae*	**26**	**12**	[32]
*Entamoeba histolytica*	**20**	**13**	[33]
*Plasmodium falciparum*	**22**	**ND**	[34]
*Leishmania major*	**28**	**18**	[35]
*Tripanosoma cruzi*	**30**	**19**	[35]
*Tripanosoma brucei*	**27**	**19**	[35]

* DEAH-box and Ski2-like families.

BLASTP analyses of the 46 *G. lamblia* SF2 helicases within the NCBI Human database presented the following ranges of identity and similarity, respectively: DEAD-box family (23–47% and 39–69%); DEAH-box family (26–39% and 42–54%); Ski2 family (28–43% and 47–63%); Swi2/Snf2 family (25–39% and 41–58%); RecQ family (25–32% and 41–50%); Rad3 family (27–35% and 47–51%). The unique UPF1 sequence presents 39% identity and 52% similarity to human UPF1. The yeast RNA helicase homologs, their predicted protein function and other features are also included in Additional file 4: Table S3 for each helicase identified in *G. lamblia*. The high sequence similarity between putatives RNA helicases from *Giardia* and the characterized homologous proteins suggest that they may have a similar function in RNA metabolism.

### The DEAD-box family

The 22 sequences identified from this family were aligned for further analysis and the nine consensus motifs described in DEAD-box RNA helicases from other organisms were found. The Open Reading Frame (ORF) GL50803_34684 lacks the N-terminal region including the Q Motif; when we performed a new database search, we found that the homologous gene GL50581_3622 from Assemblage B, isolate GS, possesses the complete N-terminal region. Thus, we used this region to search the isolate WB genome database and found the missing region at the CH991776, location 21991–22645. The final gene location was at the CH991776, 21991 – 23994 (+), and the gene coded for a 667-amino acid protein with all the nine consensus motifs of the DEAD-box subfamily, including the Q motif. This motif contains nine amino acids, which is a distinctive and characteristic feature of the DEAD-box family of helicases, and can interact with Motif I and a bound ATP [36].

**Figure 1:**
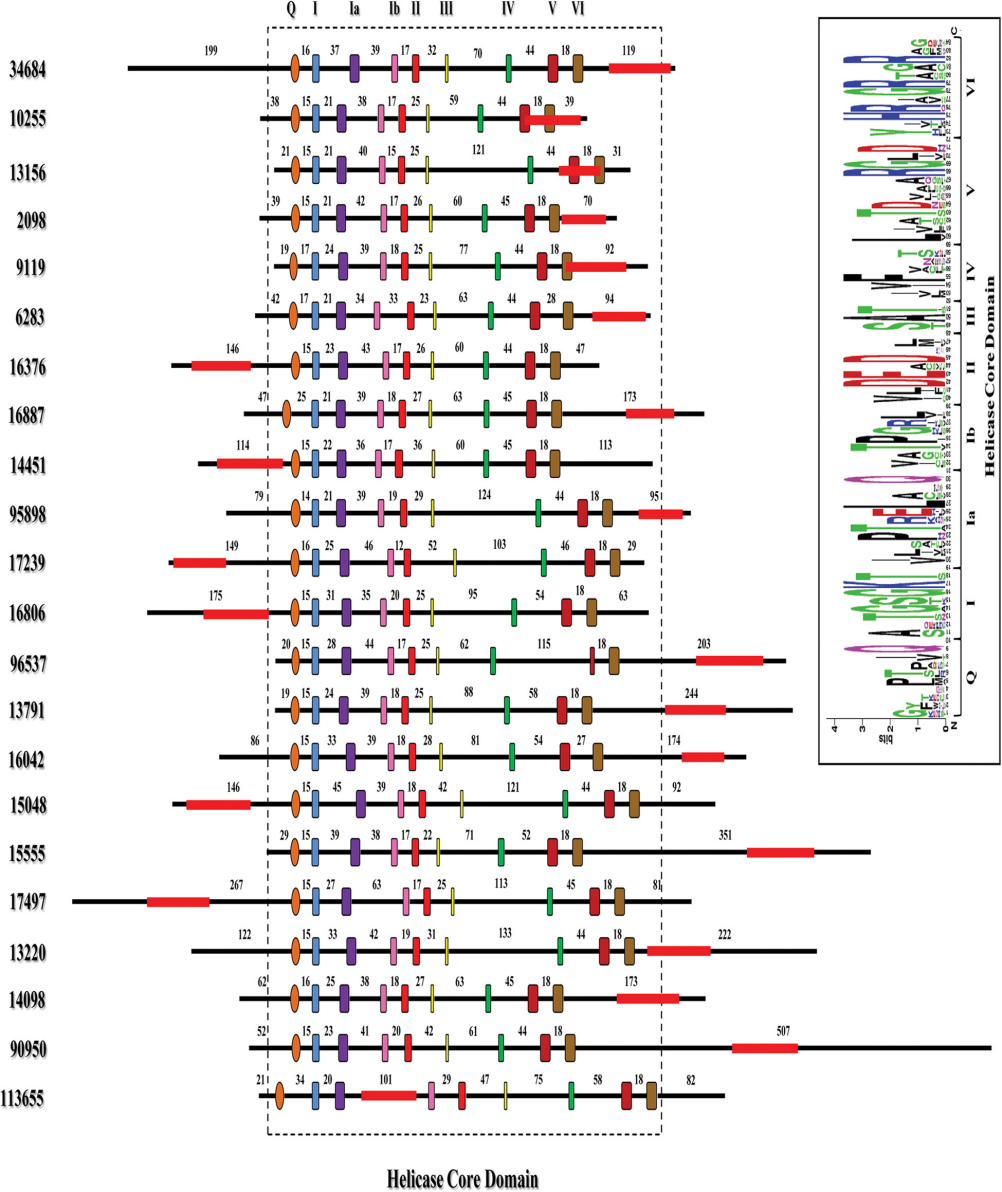
**Schematic diagram of the DEAD-box RNA helicase family in *G. lamblia *.** Each motif is represented by a different color. The distances between the motifs, and the size of the N- and C- terminal extensions for each ORF, are indicated (number of aa). The red bars within the N- or C-terminal extensions represent the regions amplified with specific primers for the qPCR. The representation is to scale. Inset: sequence LOGO view of the consensus amino acids. The height of each amino acid represents the degree of conservation. Colors mark properties of the amino acids as follows: green (polar), blue (basic), red (acidic) and black (hydrophobic).

Another characteristic amino acid, the Phe (F) residue that is close to the Q Motif was also found in 13 of the 22 enzymes, whereas in other 7 helicases, Phe (F) was replaced by Trp (W), another aromatic amino acid, being absent only in GL50803_17239 and GL50803_34684 (see Additional file 5: Figure S2). To provide a schematic graphical overview of DEAD-box sequence motif conservation, we performed a multiple sequence alignment for each motif and then used the WebLogo software to obtain a precise description of sequence similarity [37,38] (Figure 1 - inset). Analysis of regions separating each pair of consecutive motifs was consistent with the reported low sequence but high length conservation (Figure 1) [33,34]. The DEAD-box family has an N-terminal length ranging from 2 to 233 amino acids and a C-terminal length from 29 to 507 amino acids, but lack any additional domain described in other DEAD-box proteins (Figure 1) [39]. In agreement with the analyses of Banroques [40], we found that almost 55% of *Giardia* putative DEAD-box helicases have an N-terminal length of 2-45 residues and a C-terminal length of 29-95 residues, whereas the size of the HCD containing the conserved motifs ranges between 331 and 403 residues in almost 70% of this family sequences.

### The DEAH-box family

The 6 putative RNA helicases belonging to the DEAH-box family were analyzed by multiple sequence alignment and subsequent manual scanning, in search of conserved motifs characteristic of this family. As shown in Additional file 6: Figure S3, the 5 helicases present the eight characteristic motifs, with the exception of GL50803_13200, which was incomplete in its N-terminal region, missing Motif I. As with the missing motif of DEAD-box helicase GL50803_34684, a new database search showed a homologous gene, GL50581_4549 from the isolate GS, with the complete N-terminal region that was used to search the isolate WB for the entire ORF. Surprisingly, this new putative 5´ DNA genomic region does not have a traditional ATG start codon; instead, there are two putative alternative initiation codons already described in rare cases for the fungus *Candida albicans*[41] or in mammalian NAT1 [42]. Studies in progress are analyzing this finding.

**Figure 2:**
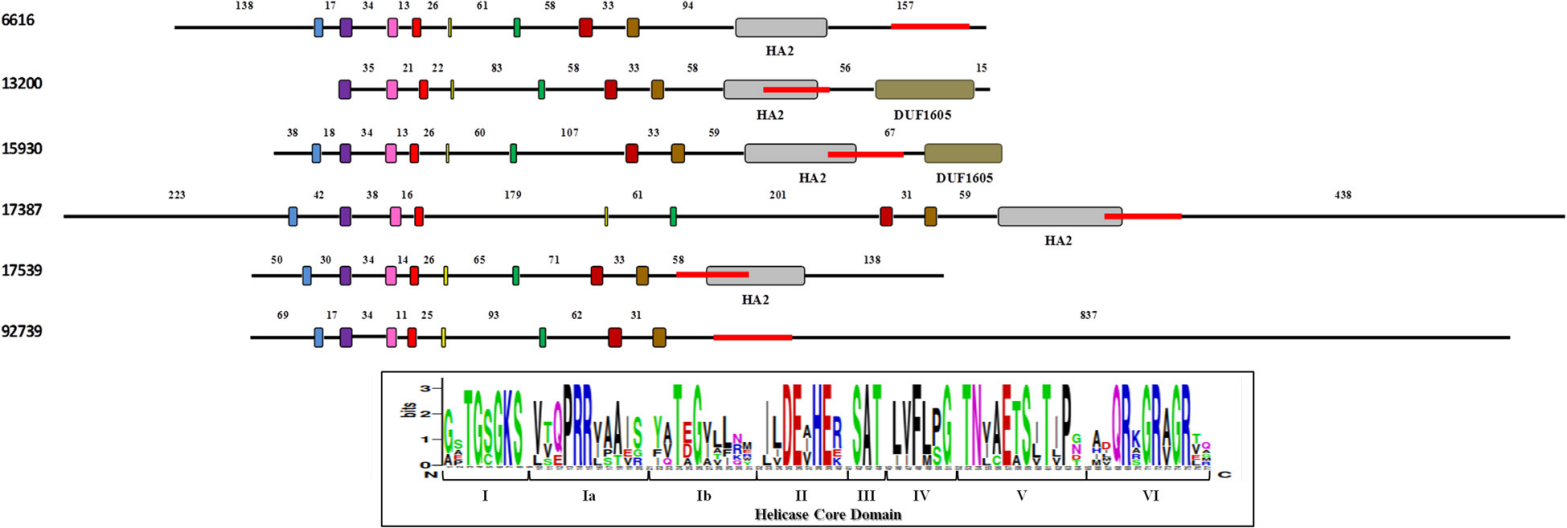
**Schematic diagram of the DEAH-box RNA helicase family in *G. lamblia. ***Each HA2 domain is represented in gray and the DUF1605 domain is represented in brown, both inside the C-terminal region. Red lines within the C-terminal extensions represent the region amplified in the qPCR for each putative helicase. The representation is to scale. Inset: sequence LOGO view of the consensus amino acids. The height of each amino acid represents the degree of conservation. Colors indicate properties of the amino acids, as follows: green (polar), blue (basic), red (acidic) and black (hydrophobic).

The consensus sequence was obtained and was in agreement with the DEAH-box motifs published by Linder and Owttrim [43] (Figure 2 - inset). Within the C-terminal regions from five of these six putative DEAH-box RNA helicases, we found another domain called Helicase Associated Domain (HA2), of about 90-120 amino acid in length (Figure 2), whose function is unknown. The HA2 domain was found in more than 1,280 eukaryotic and 590 bacterial protein sequences according to the SMART (Simple Modular Architecture Research Tool) database [44], and was present only in this DEAH-box family, being absent in all other *Giardia* putative RNA helicases. For two of these DEAH-box proteins, there was an additional domain called DUF1605 (Domain of Unknown Function).

### The Ski2 family

**Figure 3:**
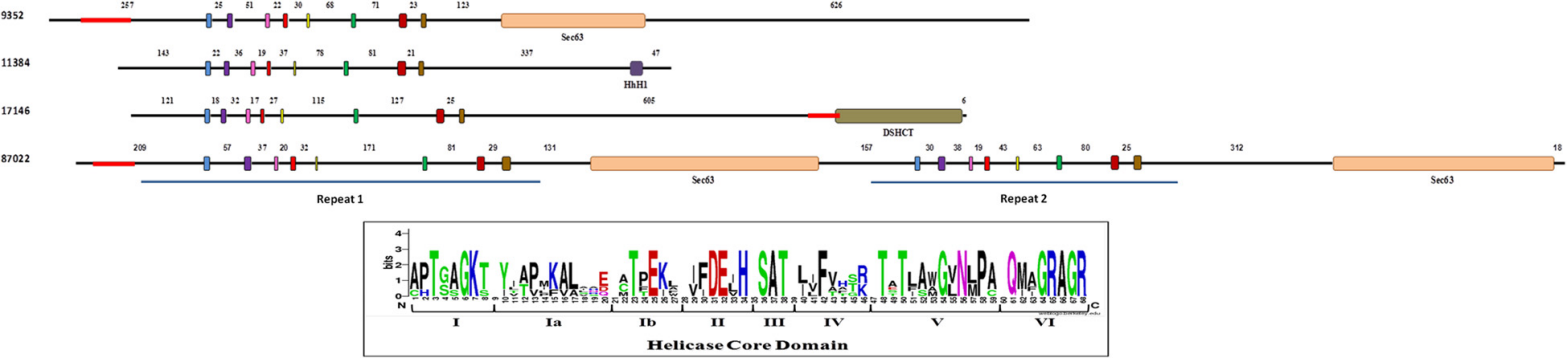
**Schematic diagram of the Ski2 RNA helicase family in *G. lamblia. *** Each Sec63 domain is represented in pink, the DsHCT domain in brown, and the HhH1 domain in violet, all inside the C-terminal region of each ORF. Red lines within the N- or C-terminal extensions represent the region amplified in the qPCR for each putative helicase. The two overlap repeats of ~ 650 amino acids are indicated in blue under the ORF 87022. The representation is to scale. Inset: sequence LOGO view of the consensus amino acids. The height of each amino acid represents the degree of conservation. Colors mark properties of the amino acids, as follows: green (polar), blue (basic), red (acidic) and black (hydrophobic).

Within this family, we found only four ORFs in the *Giardia* genome that were grouped according to the analysis of each sequence. The multiple sequence alignment (see Additional file 7: Figure S4) and the WebLogo graphic representation display the eight conserved motifs characteristic of this family [43] (Figure 3 - inset).

All of these Ski2 family members present C-terminal additional domains that can provide insights into their function (Figure 3). Two of them present a domain called Sec63, named after the yeast Sec63 protein (or NPL1) (also known as the Brl domain) where it was found, and that is required for assembly of functional endoplasmic reticulum translocons [45,46]. Another *Giardia* Ski2 protein exhibits a domain named HhH1, which is frequently found in prokaryotic and eukaryotic non-sequence-specific DNA-binding proteins [47]. The fourth Ski2 helicase presents a DSHCT domain, which is found in DOB1/SK12/helY-like helicases [48].

Interestingly, GL50803_87022 shows an internal repeat (red lines below 87022 design in Figure 3), as described for other RNA helicases [33]. This 2421-amino acid ORF consists of two RNA helicases joined together, presenting the eight Ski2 conserved motifs and an additional Sec63 motif of unknown function at the C-terminal region of each repeat. Marchat [33] detected the same patterns in several eukaryotic orthologs proteins and suggested horizontal gene transfer between bacteria and eukaryotes.

### DNA helicases from other families

As mentioned above, only six of the twelve helicase families are supposed to comprise RNA helicases (DEAD-box, DEAH-box, Ski2-like, RIG-I-like, NS3/NPH-II and Upf1-like family) and the remaining families consist of DNA helicases. In *Giardia* we found 14 additional ORFs that could be considered DNA helicases and grouped them into the three following families:

#### Swi2/Snf2 family

Seven ORFs were linked to this family based on the sequence features and compared with members of this family belonging to other species. They present the eight characteristic motifs, with the sequence conservation being represented in the logos under the alignment (see Additional file 8: Figure S5). This family is one of the largest helicase families in *G. lamblia* SF2, with an average length of 1,560 amino acids (Table S2). The N- and C-terminal regions present characteristic domains; almost all of them show one or two SNF2N domains that were described as the ATPase component of the SNF2/SWI multi-subunit complex, disrupting histone-DNA interactions. Other domains found within these ORFs were the SANT domain, the BROMO domain and a CHROMO domain.

#### RecQ family

This is the smallest family, with only three members found in the *Giardia* genome. These helicases also have one of the smallest average lengths, with only the central HCD. The eight characteristic motifs that defined this family are highly conserved, as shown in Additional file 9: Figure S6. The three ORFs share the greatest homology with the BLM (Bloom syndrome) gene from humans, which is believed to act by suppressing inappropriate recombination [49]. They are also homolog for the yeast SGS1 gene, a nucleolar DNA helicase of the RecQ family involved in genome integrity [50].

#### Rad3 family

This family is composed of four members in *G. lamblia*. It presents the largest HCD of all the SF2 helicases due to the presence of a differently large linker region between the DEXDc and the HELICc domains. They present homology in all the eight conserved motifs, except for ORF GL50803_5910, which lacks Motifs Ia and Ib (see Additional file 10: Figure S7). This ORF presents no significant similarity to human proteins; however, it was included in this family based on results of sequence and multiple alignment analyses (see Tree in Additional file 3: Figure S1).

### The helicase core domain within the dicer sequence

**Figure 4:**
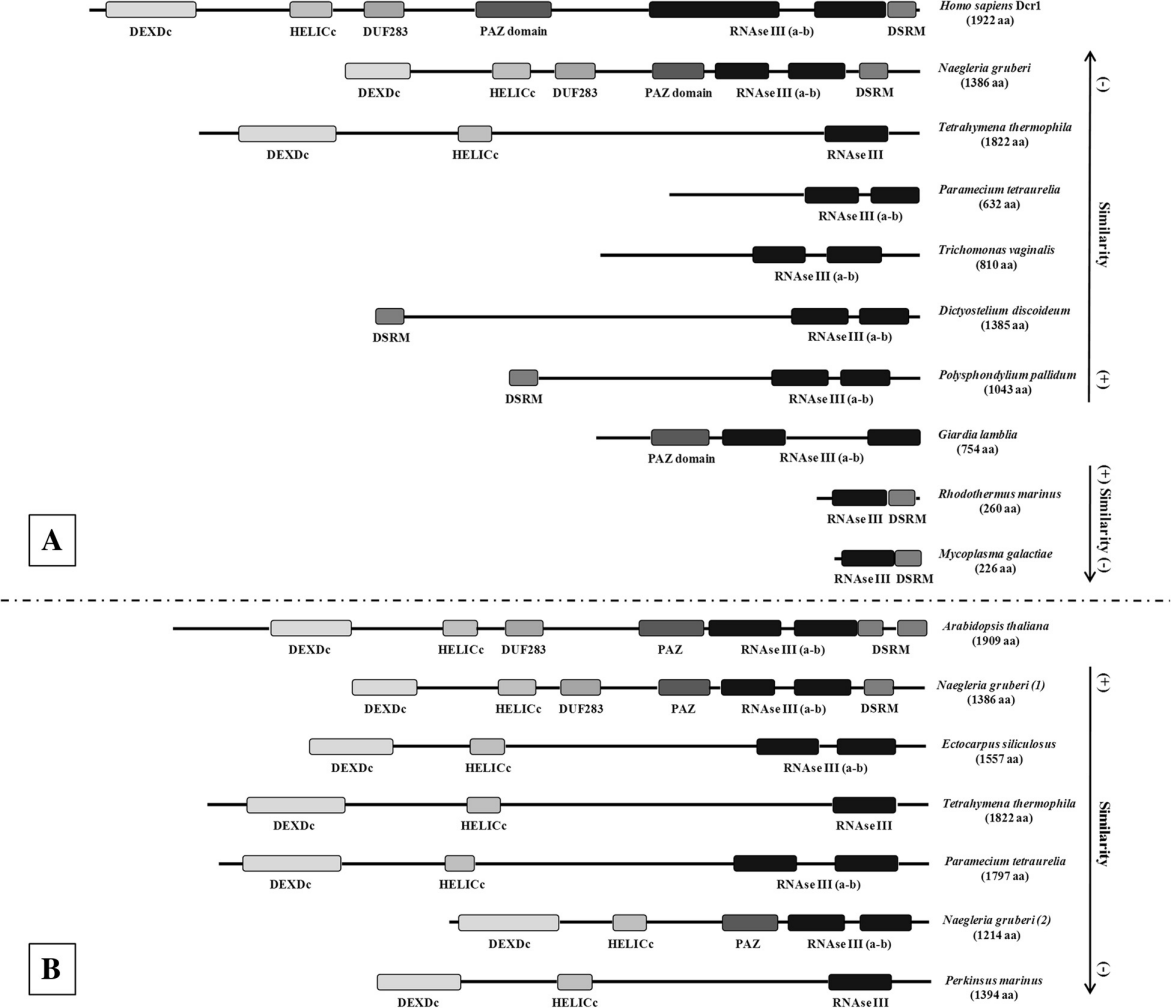
**A) Graphical representation of *Giardia lamblia *Dicer homologs.** Below the Giardia Dicer protein scheme are the two most homologous bacterial proteins found, and above it are the six protozoa most homologous proteins together with the human Dicer1 scheme. The representations are designed proportionally to their aa length, which is indicated below each organism’s name. The arrows alongside the figure indicate the degree of similarity to *Giardia* Dicer, divided into bacteria and protozoa. [Accession numbers: *H. sapiens* (Q9UPY3); *N. gruberi* (D2UZR2); *T. thermophila* (A4VD87); *P. tetraurelia* (Q3SE28); *T. vaginalis* (A2F201); *D. discoideum* (Q55FS1); *P. pallidum* (D3BF89); *G. lamblia* (A8BQJ3); *R. marinus* (D0MGH0); *M. galactiae* (D3VQS7)] **B**) Graphical representation of Arabidopsis thaliana DCL1 protozoa homologs: there are two *N. gruberi* represented in the diagram here indicated as (1) and (2). The representations are designed proportionally to their aa length, which are indicated below each name. The arrow alongside the figure indicates the degree of similarity to *Arabidopsis* Dicer. [Accession numbers: *A. thaliana* (Q9SP32); *N. gruberi*-1 (D2UZR2); *E. siliculosus* (GenBank: CBJ48587.1); *T. thermophila* (A4VD87); *tetraurelia* (Q3SD86); *N. gruberi*-2 (D2VEU9); *P. marinus* (C5LMV9)].

The HCD is an important component of higher eukaryotes’ Dicer enzymes, and is involved in some functions regarding the fundamental participation of this protein in RNAi [51-55]. As a deep-branching eukaryote, a database search using the entire *Giardia* Dicer amino acid sequence returned a list of Ribonuclease III (RIBOc) containing regions that belong to different species of bacteria with the highest alignment scores. Interestingly, these prokaryotic sequences of about 220-260 amino acids only possess one Ribonuclease III domain and one Double-stranded RNA binding motif (DSRM) (Figure 4–A).

In the search of protozoa homologs containing the HCD within the Dicer sequence, we performed a BLASTP against the protozoa genomic database available at the NCBI with the entire *Giardia* Dicer sequence. We obtained the highest score with *Polysphondylium pallidum*, which contains only an amino-terminal DSRM domain and two C-terminal RIBOc domains. The other five protozoa with the highest scores against *Giardia* Dicer protein present different domains, as shown in Figure 4–A. The homologies were located only at the C-terminal region, spanning the two conserved RIBOc domains together with the PAZ domain. Interestingly, one of these homologs from *Naegleria gruberi* presents all the conserved domains, being also the protozoa protein with the highest sequence similarity to human Dicer1 (Figure 4–A). Remarkably, the HCD of this protozoan enzyme have low homology with any putative RNA helicases found in *Giardia*, as is also the case for the well-conserved helicase domain within other higher eukaryotes Dicer proteins used to search the *Giardia* genome database.

Using the Dicer-like 1 (DCL1) protein sequence from *Arabidopsis thaliana*, we searched the protozoan database for other Dicer-like proteins that could have the HCD together with the Ribonuclease III domains. Noticeably, besides the *N. gruberi* putative protein containing all the Dicer conserved domains, other protozoan homologs that have at least these two domains are *Ectocarpus siliculosus*, *Tetrahymena thermophila*, *Paramecium tetraurelia*, and *Perkinsus marinus* (Figure 4–B). These molecules do not present all Dicer domains and, in some cases, they only show one Ribonuclease III domain instead of two. Additionally, by taking only the HCD of these protozoa proteins and performing a BLASTP against the *Giardia* assemblage A isolate WB database, we did not find any significant homology with the described putative RNA helicases. Even when we generate a profile sequence from these five protozoan (the complete sequence or just the HCD sequence) and performed a more sensitive PSI-BLAST (iteration 5), the *Giardia* sequences presented low homology and corresponded to helicases already described in this work.

We also used eight Dicer sequences from higher eukaryotes (*S. pombe*; *M. truncatula*; *H. sapiens*; *M. musculus*; *X. laevis*; *A. thaliana*; *D. melanogaster* and *C. elegans*), all of them presenting a helicase domain and almost all the others Dicer specific domains (a PAZ domain, two Ribonuclease III domains and dsRNA binding motif). Considering only their HCD, we created a consensus sequence of 613 amino acids. A PSI-BLAST analysis (iteration 5) of the *G. lamblia* database using this consensus sequence give us 39 putative helicases already described and classified in this work. The best E-value was for the DEAD-box putative helicase GL50803_95898, with query coverage of only the 30%. To analyze the presence of patterns conserved in sets within this eight helicase domains, we performed a pattern matching using the Pratt software [56]. We obtained a series of best sets and subsets patterns that could be divided into four groups, two in the DEXDc domain, one in the HELICc domain and one in the region within this two. These four patterns were used again to search the *Giardia* database. First, we created a consensus sequence for each one of these patterns and used it to perform a PSI-BLAST analysis (iteration 5). Only with the best pattern, corresponding to the HELICc domain, our analysis gave a series of similar sequences, all of them already described as putative helicases. Again the putative DEAD-box helicase GL50803_95898 was at the top-five sequences with a 100% query coverage. The other patterns obtained provided no sequences producing significant alignments with E-value better than threshold.

### RNA helicases relative expression during encystation

**Figure 5:**
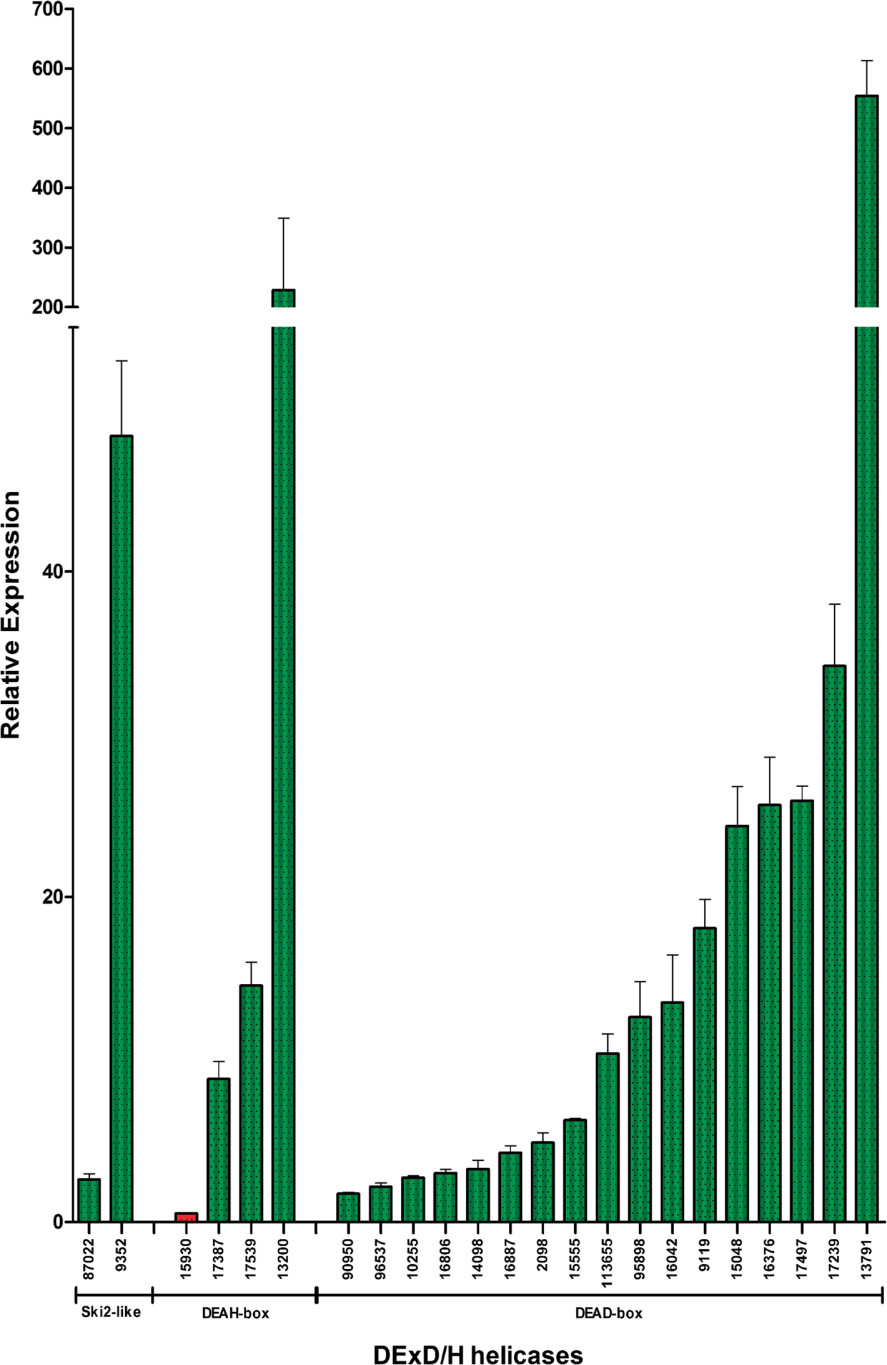
**Real time quantitative PCR (qPCR) of RNA helicases from *G. lamblia *during encystation.** The graph is a representative qPCR determination of three independent biological replicates. The ORFs are indicated at the bottom of the graph and separated in families. The up-regulated ORFs are represented in green bars, and the down-regulated ones, in red bars, each one with the corresponding relative expression ratio.

Based on *in vitro* experiments, the contribution of several DExD/H-box proteins in the accomplishment of crucial cellular functions has been revealed [30]. The fact that the entire life cycle of *G. lamblia* can be reproduced *in vitro* makes this species an attractive model to study cellular differentiation [57]. We analyzed the expression of all the DExD/H-box helicases genes by quantitative PCR (qPCR) (except for the ORF GL50803_11384 which presented very low efficiency in two pairs of primers tested), to explore the participation of these genes during the encystation process. The relative expression of these genes was determined in trophozoites under normal proliferating conditions, and in those induced to encyst after incubation for 16 hours in encystation medium, as described in Materials and Methods. Of a set of thirty one genes studied, we found eight whose expression did not change during encystation, five from the DEAD-box family, two from the DEAH-box family and one from the Ski2-like family. We also found down-regulation of one gene from the DEAH-box family after induction of trophozoites differentiation into cysts. In addition, we found twenty two genes that were up-regulated during encystation, seventeen from DEAD-box family, three from the DEAH-box family and two from the Ski2-like family (Figure 5). The encystation process was confirmed in these samples by analyzing the expression of a developmentally-regulated molecule [58] by Western blotting using a specific anti-CWP2 (Cyst Wall Protein 2) monoclonal antibody (see Additional file 11: Figure S8).

Comparing the up-regulated genes reported in the SAGE (Serial Analysis of Gene Expression) data [59] (sense tags) we found some correlation (11/21) with the DEAD-box family; (2/4) with the DEAH-box family and (1/3) with the Ski2-like family (see Additional file 12: Figure S9). The ORF GL50803_10255 was not included in the graph because the percentage of the sense tags was almost 10 times the percentage of the others ORFs in this study, but up-regulation of this gene correlated with the qPCR determination. This comparison between the qPCR results and the SAGE data should be taken with caution, as the induction protocols and the time points considered are not directly comparable. One explanation for the low agreement between the two methods is that encystation is poorly synchronic [59]. Another possible reason, as previously described for the validation process between two different methods of gene expression determination [60], is that these analyses have inherent pitfalls that may significantly influence the data obtained for each method and, in general, those genes showing small degrees of change also present lower correlations [61]. We were not able to determine the correlation of the down-regulated ORF GL50803_6616 or of the up-regulated ORF GL50803_17539 because there is no determination in the SAGE data, probably they are among the 7,256 unassigned SAGE tags [59]. We could not find also sense tag determination in the SAGE data for the ORF GL50803_113655. Taking in account the postulate that encystation genes can be divided into specific and up-regulated after induction [62], we assume that the twenty two putative RNA helicases genes with high relative expression described here would fall into the latter group.

### RNA helicase relative expression during antigenic variation

**Figure 6:**
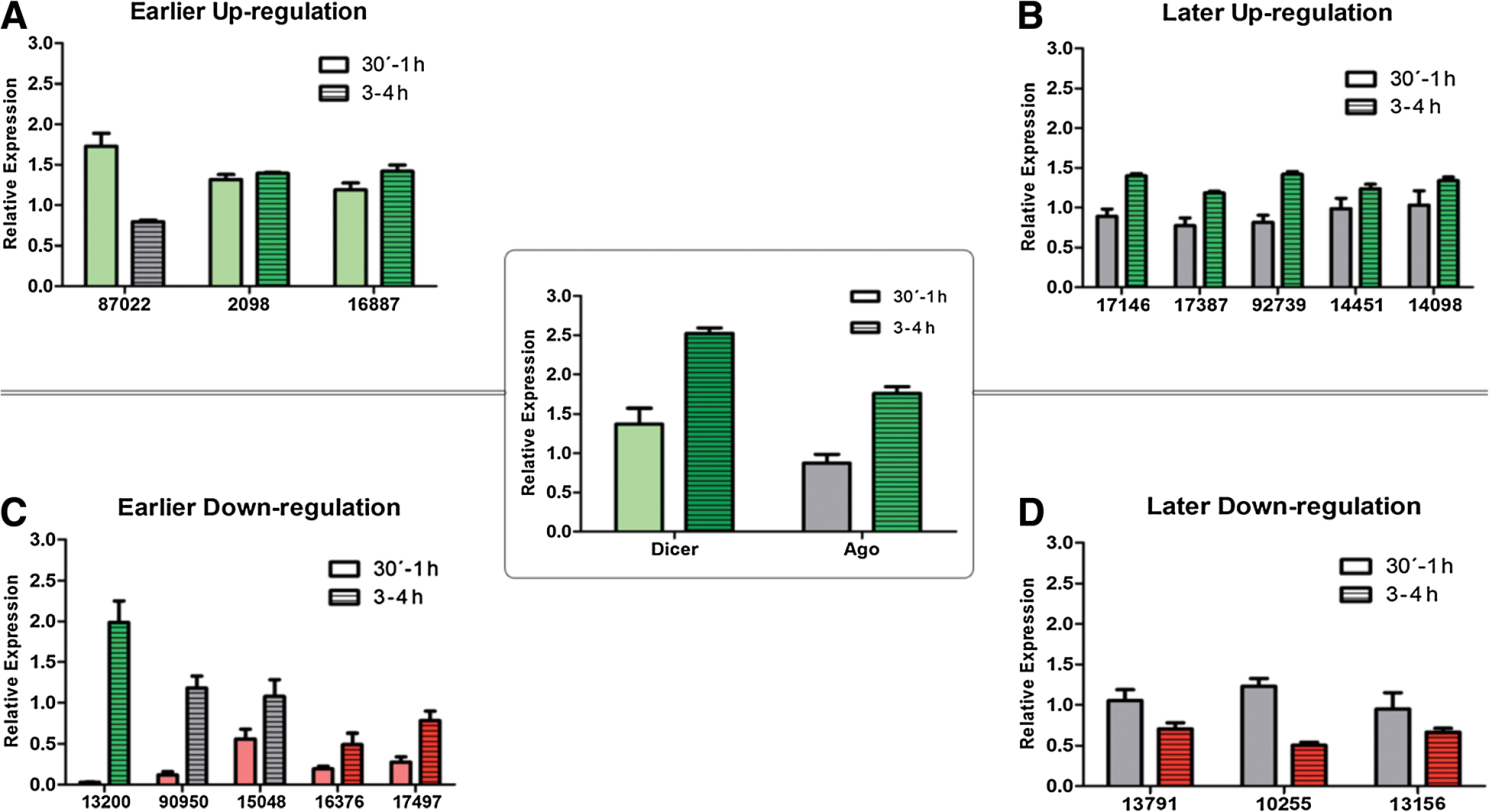
**Real time quantitative PCR (qPCR) of RNA helicases from *G. lamblia *during antigenic variation.** The relative expressions were calculated after induction of antigenic variation for 30 min – 1 hour (empty fill pattern) and for 3 to 4 hours (line fill pattern). The relative expression from different helicases was divided into up-regulated (upper panel) and down-regulated (lower panel). Green bars represent significant up-regulation and red bars represent significant down-regulation, gray bars represent no change in the relative expression. **A**. Helicases up-regulated during the first 30 min to 1 h. **B**. Helicases up-regulated at 3 to 4 h. **C**. Helicases down-regulated during the first 30 min to 1 h. **D**. Helicases down-regulated at 3 to 4 h. Center inset: relative expression for *Giardia* Dicer and Argonaute at earlier or later time points. The ORFs are indicated at the bottom of the graph. The graphs represent the mean of three different measures and the respective standard deviation.

Antigenic variation was induced on a unique VSP-expressing *Giardia* clone. The primers used for these determinations were the same as those used for the study of the encystation process. We also designed two additional pairs of primers to determine the relative expression of *Giardia* Dicer and Argonaute (Ago) transcripts. The relative expression from the thirty one *Giardia* putative RNA helicases was divided into earlier (30 min – 1 h) and later (3 – 4 h) up-regulated or down-regulated transcripts. Eight putative RNA helicases were up-regulated after antigenic variation induction, three of them earlier and five later. On the other hand, eight putative RNA helicases were down-regulated, five after early induction and three later (Figure 6).

A more detailed analysis of the relative expression of the eight putative RNA helicases that were up-regulated after antigenic variation induction showed a slight induction ranging from 1,189 to 1,729 times. In addition, two transcripts from the early up-regulation maintain induction after 3-4 hours. The eight down-regulated putative RNA helicases presented strong down-regulation earlier and significant down-regulation later during antigenic variation. Two of the five early down-regulated RNA helicases maintained low levels of expression after 3-4 h, while one of them was up regulated later. The three transcripts that were down-regulated later presented no significant variations at 30 min-1 h (Figure 6). The relative expression of gDicer presented an early up-regulation that is maintained at later times, while *Giardia* Ago presented a later up-regulation after 3-4 post induction of antigenic variation (Figure 6, inset).

Based on our results from the qPCR experiments, we searched the *Giardia* database for highly homologs to well known RNA helicases already described participating in the RNAi process in higher eukaryotes. We found that the human DEAH-box helicase RHA (DHX9), described in remodeling RISC to allow dsRNA loading onto this complex [52], has a high homology with the *G. lamblia* DEAH-box helicase GL50803_13200, which presents a later up-regulation during antigenic variation, in agreement with the *Giardia* Ago expression (3–4 h post induction). Another *G. lamblia* DEAH-box helicase found to have high homology with the HsRHA is GL50803_17387, which also presents a delayed up-regulation after induction of antigenic variation. Interestingly, a *Giardia* putative RNA helicase that presented an early up-regulation that was maintained for 3–4 h after antigenic variation induction is GL50803_2098, which has a great homology with the human DDX6 helicase (p54), a protein that interacts with Ago2 in affinity-purified RISC assemblies to facilitate formation of cytoplasmic P-bodies and that acts as a general translational repressor in human cells [63].

Other bona fide RNAi component in *D. melanogaster* S2 cells is the Belle (Bel) DEAD-box RNA helicase that seems to be important to both pathways (miRNA and siRNA). Our search found that the *G. lamblia* putative DEAD-box helicase GL50803_15048 present the highest homology with this *Drosophila* helicase described acting downstream of the dsRNA loading onto the RISC. Our qPCR data shows that even when the *Giardia* putative helicase GL50803_15048 presented an early down-regulation, their mRNA levels increased at 3–4 hs after the antigenic variation induction. The *G. lamblia* DEAD-box helicase GL50803_15048 was also found to have a high homology with two other RNA helicases described participating in the RNAi pathway. This two related DEAD-box RNA helicases (p68 and p72) were found to associate with a complex containing Drosha and required for processing of miRNA in mice [64].

Western blotting from total protein of the different samples and times analyzed by qPCR in the antigenic variation experiment showed that the level of the specific VSP protein do not change (see Additional file 13: Figure S10). Under these experiments conditions, a change in VSP protein expression was detected by immunofluorescence assays after 48 h. Since our intention was to determine the early participation of some putative helicases during this specific *Giardia* adaptation process, we performed qPCR reactions only at very short times (from 30 min to 4 h post- induction), where the changes at the protein level for VSPs cannot be detected. Although there was no VSP change at these times, we were able to detect specific up regulated expression of Dicer and Ago transcripts, two essential enzymes already related with this process [22]. Importantly, Dicer expression was up regulated at very short times and was maintained for hours, while gAgo expression raised at later times, in accordance with their roles in the RNAi process [65]. Although there is an incomplete understanding of how RNA helicases are regulated, it is possible that they operate at different steps of the RNAi pathway or performing different roles [66].

## Discussion

As shown in several studies, RNA helicases are involved in a wide variety of processes, some of them being essential for survival, as demonstrated for the yeast putative RNA helicases, where their knockouts were lethal [32]. These results are essential for the correct annotation of the *Giardia* genome, since many of the helicases identified in this study were automatically annotated either as helicases without indicating any further information and others just as hypothetical proteins (http://www.giardiadb.org).

The genome of a number of organisms contains a large number of putative helicases [34] and, as we found in this work, the relationship between the number of DEAD-box and DExH-box RNA helicases is conserved in *Giardia* as it is has been reported for other organisms (Table 1). Although *Giardia* is considered as an early-branching eukaryote and has a smaller and more compact genome [67], our findings regarding the type and number of RNA helicases in *Giardia* highlight the importance of these molecules in the biology of eukaryotic cells.

Since only a few DExD/H-box RNA helicases have been characterized biochemically, most of the reports assigning a putative function are based on the presence of the conserved and characteristic motifs that can define a putative RNA helicase and its family. Here we used the presence of those motifs for classification performing an *in silico* approach and then by manual identification of each motif. Then we confirmed and refined each motif at each position. Our results were in agreement with the phylogenetic tree obtained, because SF2 helicases were grouped specifically according to their sequence conservation as well as with the conservation of their motifs.

The particular finding within the *Giardia* Ski2 family regarding the internal duplication of the ORF GL50803_87022, having two helicases and Sec63 domains, probably indicates that the origin of this protein was by a fusion event of two ancestral prokaryotic genes, as proposed for the RNA helicases from *Entamoeba histolytica* EhDExH1 and EhDExH10 [33] and other homologous proteins from phylogenetically distant species. Unfortunately, the significance of this duplication found only in two early-branching parasitic intestinal protozoa is still unknown.

The DEAD-box protein family is present in many organisms, being the major RNA family of helicases, which seem to be involved in many, if not all, steps of RNA metabolism [68]. Although some DEAD-box helicases are closely related and have been described as paralogs [33], the comparison among amino acid sequences of all full-length sequences showed no paralogous DEAD-box helicases in *Giardia* because these proteins only share 14–29% identity and 24–43% similarity.

Regarding *Giardia* differentiation into cysts, it is known that encystation comprises the formation of a resistant cyst wall that allows the parasite to survive under hostile external environmental conditions and guarantees the transmission of the infection to susceptible hosts [69]. Several encystation-specific genes have been identified and characterized during the last decade, and have shown to be up-regulated with similar kinetics during encystation, suggesting that their regulation is at the transcriptional level [70]. Several reports also described putative transcription factors that regulate the expression of encystation-specific genes [71-74]. It was assumed that the encystation process is controlled at multiple levels (basic transcription, enhancement or de-repression) [62]. Moreover, it was hypothesized that epigenetic chromatin modifications via histone acetylation/deacetylation may participate in modulation of stage differentiation in this parasite [75]. In higher organisms, different RNA helicases have been described to interact with histone deacetylases (HDACs), such as the known transcriptional regulator DP103 (Ddx20, Gemin3), which was found to immunoprecipitate with histone deacetylases HDAC2 and HDAC5, suggesting a role in transcription repression through HDACs recruitment [76]. In addition, the role of the RNA helicases p68 (Ddx5) and p72 (Ddx17) as transcription repressors when interacting with HDAC1 [77], HDAC2 and HDAC3 has been reported [78]. Our findings regarding the levels of induction of the RNA helicase genes by qPCR were diverse, ranging from a smooth 2-4-fold induction in some DEAD-box genes to a high (20-31 times) relative expression in other genes. Two genes, DEAD-box GL50803_13791 and DEAH-box GL50803_13200, presented a marked induction of 554 and 228 times, respectively, under the encystation conditions. Notably, the up-regulation of the encystation-specific gene coding for CWP2 increased up to 2,187 times compared to its expression in trophozoites.

In *Giardia*, the RNAi machinery controlling antigenic variation has been found to involve a Dicer enzyme with unique characteristics when compared to Dicer enzymes from higher eukaryotes. *Giardia* Dicer lacks the DExD/H helicase domain as well as double-stranded RNA binding motifs present in other Dicer homologs. Because we are only starting to understand the different roles of RNA helicases in RNAi, there are still many unresolved questions. Since different RNA helicases might operate at different steps in the RNAi pathway or might play different roles, the presence of thirty two putative DExD/H-box helicases in the *Giardia* genome and their differential patterns of expression during antigenic variation support their importance for RNAi. It would be relevant to determine the role of particular *Giardia* RNA helicases for different subsets of miRNA or siRNAs. However, it is already clear that the presence of a RNA helicase activity (unwinding or as adaptor proteins) is necessary for the correct functioning at different steps of the RNAi pathway. As it was proposed in several reports, there are a number of potential roles for RNA helicases in RNAi [66]. Our findings in the qPCR experiments during antigenic variation suggest that RNA helicases may participate in RNAi. This could be the case of the *G. lamblia* putative DEAD-box helicase GL50803_15048, which was found to present high homology with the DmBel helicase and also with the DEAD-box RNA helicases p68 and p72. Taking into account that some studies pointed out extensive overlapping and interplay among small RNA directed silencing machineries [64] and different RNA helicases operate either at different steps or playing different roles in the RNAi pathway, the involvement of this *G. lamblia* RNA helicase (GL50803_15048) in post-transcriptional gene silencing deserve further analysis.

Although we did not find a putative helicase in *Giardia* with high similarity to the HCD of higher eukaryotes Dicer enzyme, it has been proposed that Dicer helicase domain is required for siRNA, but not miRNA, processing [79]. Point mutations within the helicase domain or Dicer lacking a functional HCD showed that pre-miRNA processing does not require helicase participation, but that it is necessary for long dsRNA (siRNA processing) [79].

In *Giardia*, we have demonstrated that purified RdRP generates high-molecular-weight VSP RNAs *in vitro* only when more than one VSP transcript is present in the reaction mixture [22] and proposed a mechanism where variations in either the general or local concentrations of different VSP transcripts may determine which transcript will circumvent the silencing system, as was suggested to occur in higher eukaryotes [53]. In addition, it has been proposed by others groups the presence in *Giardia* of a miRNA biogenesis pathway reminiscent of the canonical miRNA biogenesis pathway found in higher organisms [25,80], and they have identified conserved putative microRNA target site of several variant surface protein (VSP) mRNAs. Here *Giardia* Dicer apparently would assume the functions of both a Drosha and a Dicer, although no RNA-binding protein DAWDLE (DDL) homolog has yet been identified in this parasite. Furthermore *Giardia* Dicer must shuttle between the cytoplasm and the nucleus to process pri- and pre-miRNAs, although we determined its cellular localization by expressing a hemagglutinin-tagged version of the protein. Similar to that observed in other cells, *Giardia* Dicer localizes to the cytoplasm [22].

On one hand, the lack of the RNA helicase domain in *Giardia* Dicer is in agreement with the occurrence of a miRNA pathway. But, on the other hand, it was also proposed that a deletion or mutation of the helicase domain of human Dicer leads to a more active enzyme *in vitro* for cleavage of a perfectly matched 37-nt linear duplex RNA [51], allowing the enzyme to rapidly reinitiate cleavage on the long substrates. This last being the case for the generation of perfectly matched VSP dsRNAs by RdRP and subsequent degradation [22]. It is also important to highlight that the lack of the helicase domain was proposed to increase the effectiveness of long hairpins for intracellular applications in which multiple siRNAs are desired, as could be the case for VSP mRNA degradation. Interestingly, gDicer without the RNA helicase domain can complement the absence of the entire Dicer in *S. pombe*[26]. The lack of the RNA helicase domain in *Giardia* Dicer or, in other words, the inclusion of the RNA helicase domain in Dicer enzymes of higher eukaryotes, raises new questions about the function of this domain in Dicer activity and regulation.

## Conclusions

The first *in silico* classification of SF2 *G. lamblia* helicases was achieved, describing some of their features, organization, structure, and homology to helicases from humans and yeast. A series of up- and down-regulated putative RNA helicases were found during encystation and antigenic variation, suggesting their participation in both adaptative processes. Most of them are assumed to be up-regulated after induction to encystation, while in the antigenic variation process we infer that the regulated RNA helicases studied may operate at different steps of the RNAi pathway, even when no putative helicase in *Giardia* presented high similarity to the HCD of higher eukaryotes Dicer enzymes.

## Methods

### Screening of databases

The *G. lamblia* complete genome sequence was screened at the *Giardia* Genome Resource [28] (strain ATCC 50803, Assemblage A, isolate WB) using the PSI-BLASTP program. The query used was the complete amino acid sequence of the human Eukaryotic Initiation Factor 4A-I (eIF4A) and the human ATP-dependent RNA helicase DHX8 as DEAD and DEAH-box prototypes, respectively. For the determination of identity/homology sequences within the human genome, we performed a BLASTP search at the NCBI Human database using the default parameters and the Build protein database. The yeast homologous proteins were obtained with the HomoloGene option from the NCBI database according to the human RNA helicase previously found, and the gene functions or characteristics are based on the literature. For the Helicase Core Domain analysis, we performed a BLASTP search using the entire *Giardia* Dicer amino acid sequence (ORF GL50803_103887). One search was conducted within the entire NCBI proteins database and the other only within the protozoa database available at the NCBI BLAST Assembled RefSeq Genomes. The search of protozoa proteins homologous to the *Arabidopsis thaliana* Dicer-like 1 was performed within the protozoa database at the NCBI website. The similarity between the Helicase Core Domain of the protozoa proteins found and the *Giardia* database was performed at the *Giardia* Genome Resource (strain ATCC 50803, Assemblage A, isolate WB) using the BLASTP program.

### Sequence analysis

Multiple sequence alignment was performed with the ClustalW2 program at the European Bioinformatics Institute (EBI). For the alignments of each RNA helicase family shown in the figures we used the Multiple Align Show at The Sequence Manipulation Suite, specifying the fraction of residues that need to be identical or similar in a column of the alignment at 70% for highlighting. For the “Ident and Sim” analysis within the DEAD-box sequences, we first performed a MUSCLE alignment at the EBI website and then ran the program at “The Sequence Manipulation Suite”. The structural domains and sequence patterns were first predicted at the Eukaryotic Linear Motif resource (ELM) [81], getting the DEXDc and HELICc RNA helicase domains and the HA2 and Sec63 domains. After that, each specific family motif was checked manually and indicated using the putative consensus motifs described in the literature [43]. For the graphical representation of the amino acid conserved motifs within each family we used the web-based application WebLogo [38], where each logo consists of stacks of symbols, one stack for each position in the sequence. The overall height of the stack indicates the sequence conservation at that position, whereas the height of symbols within the stack indicates the relative frequency of each amino or nucleic acid at that position. The putative Dicer amino acid sequence analysis was performed using the Eukaryotic Linear Motif resource (ELM) and the ExPASy - PROSITE database [82].

### Phylogenetic analysis

We used only the helicase domain from the RNA helicases selected to run a multiple alignment (MUSCLE) into the SeaView Version 4.2.12 [83-86]. Then we computed the tree using PhyML v3.0.1 as an external program [86].The design was edited using the Tree Figure Drawing Tool Version 1.3.1.

### Cultures

*G. lamblia* trophozoites were cultured in TYI-S-33 medium at pH7.0 with 10% adult bovine serum and bovine bile (0.5 mg/ml) [87] in anaerobiosis at 37°C. For induction of encystation, the trophozoites were cultured until confluence and then the medium was replaced with encystation medium (porcine bile 0.45%, lactic acid 0.01% and pH 7.8) [88] and grown in anaerobiosis at 37°C during 16 h. For antigenic variation experiments, a *Giardia* clone expressing VSP-1267 was obtained by serial dilution and selection by immunofluorescence assays using specific monoclonal antibody that recognizes only this VSP, and then cultured until 90% confluence. Induction of antigenic variation was performed according to Torri et al. (manuscript in preparation).

### RNA extraction and cDNA synthesis

Total RNA was extracted from each sample (trophozoites and encystation induction) using Trizol reagent (Invitrogen) according with manufacturer’s instructions. Total RNA was spectrophotometrically quantified and treated with DNase I (Roche) at 37°C for 1 h. After DNase inactivation total RNA was quantified again and several PCRs were performed to check for the presence of genomic DNA. If no DNA was detected after PCR, we performed the cDNA synthesis using SuperScript III Reverse Transcriptase (Invitrogen), following manufacturer’s instructions. All newly synthesized cDNA were collected together for the subsequently qPCR reactions.

### Quantitative real time PCR (q-PCR) of RNA helicase mRNA

Quantitative PCR was performed using the QuantiTect SYBR Green PCR kit (Qiagen). We used 1 μl of cDNA in a final volume of 25 μl; a triplicate for each gene was performed. The primers used for this determination (0.6 μM each) were designed based on the N- or C-terminal extensions because they are highly variable in size and composition, and have no significant homology between them, making every pair of primers specific for each helicase as shown in Figures 2, 3 and 4 (red bars). Thermal conditions were as follow: initial incubation for 15 min at 95°C, 15 sec at 95°C, 30 sec at 50°C and 30 sec at 72°C for 35 cycles, with the plate read after each cycle, and a final incubation for 10 min at 72°C. The Melting Curve was performed from 50°C to 90°C, with a plate read at every 1°C. We used the Chromo4 system for Real-time PCR detection (BioRad) and the data collected was analyzed using the REST 2009 (Relative Expression Software Tool V2.0.13 – Qiagen) [89]. RNA was standardized by quantification of glutamate dehydrogenase (gdh) as a reference gene.

### Protein isolation and Western blot analysis

Total protein extraction was performed from the same Trizol extraction procedure, as indicated by the manufacturer. Total protein content was determined with the BCA™ Protein Assay kit (Pierce). Fifty micrograms of total protein was loaded onto a 10% polyacrylamide gel (SDS-PAGE) and after running, it was transferred to a PVDF membrane (Immobilon–P, Millipore). The membrane was blocked with 5% milk in TBS-Tween20 for 1 hour and then incubated with a monoclonal antibody (mAbs 7D6) specific against *G. lamblia* CWP2 [1:2000]. After three washes with TBS-Tween20, the membrane was incubated with goat anti-mouse immunoglobulin serum conjugated with alkaline phosphatase [1:2000] (Southern Biotechnology) and revealed with alkaline phosphatase substrate (BCIP/NBT, Color Development Solution, BioRad).

### Accession numbers

See Additional file 14: Table S4 for a complete list of proteins cited in the manuscript, organism it is derived and NCBI reference sequence number.

## Abbreviations

SF: Superfamilies; HCD: Helicase Core Domain; VSP: Variant-specific Surface Protein; RNAi: RNA interference; RHA: RNA helicase A; Bel: Belle; Armi: Armitage; eIF4A: Eukaryotic Initiation Factor 4A; ORF: Open Reading Frame; HA2: Helicase-Associated Domain; SMART: Simple Modular Architecture Research Tool; DUF1605: Domain of Unknown Function; CHROMO: CHRromatin Organization Modifier; BLM: Bloom syndrome; RIBOc: Ribonuclease III; DSRM: Double-stranded RNA binding motif; DCL1: Dicer-like 1; CWP2: Cyst Wall Protein 2; qPCR: Quantitative PCR; SAGE: Serial Analysis of Gene Expression; ELM: Eukaryotic Linear Motif; gdh: Glutamate dehydrogenase.

## Competing interests

The authors declare that they have no competing interests.

## Authors’ contributions

PRG performed bioinformatics and sequence searching and comparison analysis, including motif and phylogenetic analyses, and assisted with manuscript writing. MCS performed the qPCR experiments, including the production of *G. lamblia* cultures. AT performed the induction of encystation and antigenic variation. HDL coordinated the project, writing process and analyses. All the authors read and approved the final manuscript. HDL is Guggenheim Fellow; PRG and HDL are Members of the Scientific Investigator’s Career of the National Research Council of Argentina (CONICET). All authors read and approved the final manuscript.

## Supplementary Material

Additional file 1: Table S1 Putative SF2 Helicases from *Giardia lamblia.* The table indicates the Family, the gene number from the Assemblage A isolate WB (the number that is given should be preceded by the prefix GL50803_), the current Supercontig or positions where it is located, the number of nucleotides in base pairs (bp) and molecular mass of the putative protein in kDa, for each putative helicase. (DOCX 18 kb) Click here for file

Additional file 2: Table S2 Average lengths (amino acid) of SF2 helicase families from *Giardia lamblia.* The table indicates the average length (in number of amino acids) of each SF2 helicase family. The incompletes sequences were not considered in the computation. Click here for file

Additional file 3: Figure S1 Phylogenetic tree of the 46 putative SF2 helicase genes in *Giardia lamblia.* Phylogenetic tree derived from the alignment of the “Helicase Core Domain” amino acid sequences. Each helicase is named after its gene number, as in the GiardiaDB. The family groups are indicated as follows: DEAD-box (orange), DEAH-box (green), Ski2 (violet), RecQ (pink), Swi2/Snf2 (light orange) and Rad3 (light blue). Click here for file

Additional file 4: Table S3 *Giardia lamblia* SF2 helicases homologues in human and yeast. The table indicates each putative *Giardia* helicase with its Accession Number and ORF, the protein length in aminoacid, its putative helicase homologue form human with the identity and similarity percentage, and its putative helicase homologue from yeast with their known functions. Click here for file

Additional file 5: Figure S2 Alignment of conserved DEAD-box helicase motifs. The sequences were aligned using the “Multiple Align Show” software at “The Sequence Manipulation Suite” (http://www.bioinformatics.org/sms/index.html). The residues conserved at 70% or more are highlighted in dark; other similar residues within each column are highlighted in grey. Click here for file

Additional file 6: Figure S3 Alignment of conserved DEAH-box helicase motifs. The sequences were aligned using the “Multiple Align Show” as before. The residues conserved at 70% or more are highlighted in dark; other similar residues within each column are highlighted in grey. Click here for file

Additional file 7: Figure S4 Alignment of conserved Ski2 helicase motifs. The sequences were aligned using the “Multiple Align Show” as before. The residues conserved at 70% or more are highlighted in dark; other similar residues within each column are highlighted in grey. Click here for file

Additional file 8: Figure S5 Schematic diagram of the Swi2-Snf2 helicase family in *G. lamblia.* The SANT domain is represented in blue, the BROMO domain in brown, and the CHROMO domain in green. The SNF2N domains are represented in light grey, inside each one of them are the helicase motifs, when appropriate. The representation is to scale. Inset: sequence LOGO view of the consensus amino acids. The height of each amino acid represents the degree of conservation. Colors indicate properties of the amino acids, as follows: green (polar), blue (basic), red (acidic) and black (hydrophobic). Click here for file

Additional file 9: Figure S6 Schematic diagram of the RecQ helicase family in *G. lamblia.* The representation is to scale. Inset: sequence LOGO view of the consensus amino acids. The height of each amino acid represents the degree of conservation. Colors mark properties of the amino acids as: green (polar); blue (basic); red (acidic) and black (hydrophobic). Click here for file

Additional file 10: Figure S7 Schematic diagram of the Rad3 helicase family in *G. lamblia.* The representation is to scale. Inset: sequence LOGO view of the consensus amino acids. The height of each amino acid represents the degree of conservation. Colors indicate properties of the amino acids, as follows: green (polar), blue (basic), red (acidic) and black (hydrophobic). Click here for file

Additional file 11: Figure S8 Western blot of trophozoites grown under proliferating conditions and after induction to encyst. Total protein extracts from trophozoites grown under normal proliferating conditions (Normal) or after 16hs induction in encystation medium (Encyst) were separated using a 10% SDS-polyacrylamide gel and transferred to a PVDF membrane. The membrane was incubated with a monoclonal antibody against CWP2. The iqual loading of the samples is shown in the figure at the right with a Ponceau S staining. The numbers indicate the molecular weight of protein standards in kDa. Click here for file

Additional file 12: Figure S9 SAGE (Serial Analysis of Gene Expression) data. The graph represents the sense tag percentage from *Giardia* trophozoites (white bar) and four different encystation times (4, 12, 21 and 42 hours; grayscale bars). Under each ORF it is indicated if these ORFs were up-regulated (green up arrow), down-regulated (red down arrow) or remained unmodified (equal sign). A line graph is also provided for a better identification of the expression pattern. The colored boxes represent our RT-qPCR results (with the same color code), divided into families. The asterisk under each box stands for a correlation between the SAGE and the RT-qPCR data. Click here for file

Additional file 13: Figure S10 Western blot during antigenic variation induction. Trophozoites were incubated for the indicated times with a 1:10.000 dilution of mAb 5C1directed against VSP-1267, mAb 7D2 against Cyst Wall Protein 2 or without antibody (Control). Total protein was electrophoresed, transferred to a PVDF membrane and incubated with a mAb against the VSP-1267. The molecular weights of standards are indicated in kDa. Click here for file

Additional file 14: Table S4 Accession numbers. The table indicates a complete list of proteins cited in the manuscript, the organism it is derived and the NCBI Reference Sequence Number. Click here for file

## References

[B1] Abdelhaleem M Helicases: an overview Methods Mol Biol 2010 587 1 12 20225138 10.1007/978-1-60327-355-8_1

[B2] Linder P Jankowsky E From unwinding to clamping - the DEAD box RNA helicase family Nat Rev Mol Cell Biol 2011 12 505 516 10.1038/nrm3154 21779027

[B3] Singleton MR Dillingham MS Wigley DB Structure and mechanism of helicases and nucleic acid translocases Annu Rev Biochem 2007 76 23 50 10.1146/annurev.biochem.76.052305.115300 17506634

[B4] Kainov DE Tuma R Mancini EJ Hexameric molecular motors: P4 packaging ATPase unravels the mechanism Cell Mol Life Sci 2006 63 1095 1105 10.1007/s00018-005-5450-3 16505972 PMC11136089

[B5] Rocak S Linder P DEAD-box proteins: the driving forces behind RNA metabolism Nat Rev Mol Cell Biol 2004 5 232 241 10.1038/nrm1335 14991003

[B6] Iost I Dreyfus M DEAD-box RNA helicases in Escherichia coli Nucleic Acids Res 2006 34 4189 4197 10.1093/nar/gkl500 16935881 PMC1616957

[B7] Gorbalenya AE Koonin EV Helicases: amino acid sequence comparisons and structure-function relationships Current Opinion in Structural Biology 1993 3 419 429 10.1016/S0959-440X(05)80116-2

[B8] Fairman-Williams ME Guenther UP Jankowsky E SF1 and SF2 helicases: family matters Curr Opin Struct Biol 2010 20 313 324 10.1016/j.sbi.2010.03.011 20456941 PMC2916977

[B9] Wang Y Guthrie C PRP16, a DEAH-box RNA helicase, is recruited to the spliceosome primarily via its nonconserved N-terminal domain RNA 1998 4 1216 1229 10.1017/S1355838298980992 9769096 PMC1369694

[B10] Hall MC Matson SW Helicase motifs: the engine that powers DNA unwinding Mol Microbiol 1999 34 867 877 10.1046/j.1365-2958.1999.01659.x 10594814

[B11] Bernstein E Caudy AA Hammond SM Hannon GJ Role for a bidentate ribonuclease in the initiation step of RNA interference Nature 2001 409 363 366 10.1038/35053110 11201747

[B12] Jankowsky E Fairman ME RNA helicases–one fold for many functions Curr Opin Struct Biol 2007 17 316 324 10.1016/j.sbi.2007.05.007 17574830

[B13] Edlind TD Chakraborty PR Unusual ribosomal RNA of the intestinal parasite Giardia lamblia Nucleic Acids Res 1987 15 7889 7901 10.1093/nar/15.19.7889 3118329 PMC306315

[B14] Sogin ML Gunderson JH Elwood HJ Alonso RA Peattie DA Phylogenetic meaning of the kingdom concept: an unusual ribosomal RNA from Giardia lamblia Science 1989 243 75 77 10.1126/science.2911720 2911720

[B15] Van Keulen H Gutell RR Gates MA Campbell SR Erlandsen SL Jarroll EL Kulda J Meyer EA Unique phylogenetic position of Diplomonadida based on the complete small subunit ribosomal RNA sequence of Giardia ardeae, G. muris, G. duodenalis and Hexamita sp FASEB J 1993 7 223 231 8422968 10.1096/fasebj.7.1.8422968

[B16] Hashimoto T Nakamura Y Nakamura F Shirakura T Adachi J Goto N Okamoto K Hasegawa M Protein phylogeny gives a robust estimation for early divergences of eukaryotes: phylogenetic place of a mitochondria-lacking protozoan Giardia lamblia. Mol Biol Evol 1994 11 65 71 10.1093/oxfordjournals.molbev.a040093 8121287

[B17] Feng JM Sun J Xin DD Wen JF Comparative analysis of the 5S rRNA and its associated proteins reveals unique primitive rather than parasitic features in Giardia lamblia PLoS One 2012 7 e36878 10.1371/journal.pone.0036878 22685540 PMC3369914

[B18] Adam RD Biology of Giardia lamblia Clin Microbiol Rev 2001 14 447 475 10.1128/CMR.14.3.447-475.2001 11432808 PMC88984

[B19] Lujan HD Mowatt MR Nash TE Mechanisms of Giardia lamblia differentiation into cysts Microbiol Mol Biol Rev 1997 61 294 304 9293183 10.1128/mmbr.61.3.294-304.1997 PMC232612

[B20] Nash TE Surface antigenic variation in Giardia lamblia Mol Microbiol 2002 45 585 590 10.1046/j.1365-2958.2002.03029.x 12139606

[B21] Davids BJ Reiner DS Birkeland SR Preheim SP Cipriano MJ McArthur AG Gillin FD A new family of giardial cysteine-rich non-VSP protein genes and a novel cyst protein PLoS One 2006 1 e44 10.1371/journal.pone.0000044 17183673 PMC1762436

[B22] Prucca CG Slavin I Quiroga R Elias EV Rivero FD Saura A Carranza PG Lujan HD Antigenic variation in Giardia lamblia is regulated by RNA interference Nature 2008 456 750 754 10.1038/nature07585 19079052

[B23] Rivero FD Saura A Prucca CG Carranza PG Torri A Lujan HD Disruption of antigenic variation is crucial for effective parasite vaccine Nat Med 2010 16 551 557 551p following 557 10.1038/nm.2141 20418884

[B24] Prucca CG Lujan HD Antigenic variation in Giardia lamblia Cell Microbiol 2009 11 1706 1715 10.1111/j.1462-5822.2009.01367.x 19709056

[B25] Li W Saraiya AA Wang CC Gene regulation in Giardia lambia involves a putative microRNA derived from a small nucleolar RNA PLoS Negl Trop Dis 2011 5 e1338 10.1371/journal.pntd.0001338 22028939 PMC3196473

[B26] Macrae IJ Zhou K Li F Repic A Brooks AN Cande WZ Adams PD Doudna JA Structural basis for double-stranded RNA processing by Dicer Science 2006 311 195 198 10.1126/science.1121638 16410517

[B27] Tanner NK Linder P DExD/H box RNA helicases: from generic motors to specific dissociation functions Mol Cell 2001 8 251 262 10.1016/S1097-2765(01)00329-X 11545728

[B28] Aurrecoechea C Brestelli J Brunk BP Carlton JM Dommer J Fischer S Gajria B Gao X Gingle A Grant G GiardiaDB and TrichDB: integrated genomic resources for the eukaryotic protist pathogens Giardia lamblia and Trichomonas vaginalis Nucleic Acids Res 2009 37 D526 530 10.1093/nar/gkn631 18824479 PMC2686445

[B29] Chen YH Su LH Huang YC Wang YT Kao YY Sun CH UPF1, a conserved nonsense-mediated mRNA decay factor, regulates cyst wall protein transcripts in Giardia lamblia PLoS One 2008 3 e3609 10.1371/journal.pone.0003609 18974834 PMC2572189

[B30] Umate P Tuteja N Tuteja R Genome-wide comprehensive analysis of human helicases Commun Integr Biol 2011 4 118 137 21509200 10.4161/cib.4.1.13844 PMC3073292

[B31] Umate P Tuteja R Tuteja N Genome-wide analysis of helicase gene family from rice and Arabidopsis: a comparison with yeast and human Plant Mol Biol 2010 73 449 465 10.1007/s11103-010-9632-5 20383562

[B32] de la Cruz J Kressler D Linder P Unwinding RNA in Saccharomyces cerevisiae: DEAD-box proteins and related families Trends Biochem Sci 1999 24 192 198 10.1016/S0968-0004(99)01376-6 10322435

[B33] Marchat LA Orozco E Guillen N Weber C Lopez-Camarillo C Putative DEAD and DExH-box RNA helicases families in Entamoeba histolytica Gene 2008 424 1 10 10.1016/j.gene.2008.07.042 18760338

[B34] Tuteja R Pradhan A Unraveling the 'DEAD-box' helicases of Plasmodium falciparum Gene 2006 376 1 12 10.1016/j.gene.2006.03.007 16713133 PMC7127577

[B35] Gargantini PR Lujan HD Pereira CA In silico analysis of trypanosomatids' helicases FEMS Microbiol Lett 2012 335 123 129 10.1111/j.1574-6968.2012.02644.x 22835260

[B36] Cordin O Tanner NK Doere M Linder P Banroques J The newly discovered Q motif of DEAD-box RNA helicases regulates RNA-binding and helicase activity EMBO J 2004 23 2478 2487 10.1038/sj.emboj.7600272 15201868 PMC449782

[B37] Schneider TD Stephens RM Sequence logos: a new way to display consensus sequences Nucleic Acids Res 1990 18 6097 6100 10.1093/nar/18.20.6097 2172928 PMC332411

[B38] Crooks GE Hon G Chandonia JM Brenner SE WebLogo: a sequence logo generator Genome Res 2004 14 1188 1190 10.1101/gr.849004 15173120 PMC419797

[B39] Umate P Tuteja R Tuteja N Architectures of the unique domains associated with the DEAD-box helicase motif Cell Cycle 2010 9 4228 4235 10.4161/cc.9.20.13635 20935500

[B40] Banroques J Cordin O Doere M Linder P Tanner NK Analyses of the functional regions of DEAD-Box RNA "helicases" with deletion and chimera constructs tested in vivo and in vitro J Mol Biol 2011 413 451 472 10.1016/j.jmb.2011.08.032 21884706

[B41] Abramczyk D Tchorzewski M Grankowski N Non-AUG translation initiation of mRNA encoding acidic ribosomal P2A protein in Candida albicans Yeast 2003 20 1045 1052 10.1002/yea.1020 12961752

[B42] Takahashi K Maruyama M Tokuzawa Y Murakami M Oda Y Yoshikane N Makabe KW Ichisaka T Yamanaka S Evolutionarily conserved non-AUG translation initiation in NAT1/p97/DAP5 (EIF4G2) Genomics 2005 85 360 371 10.1016/j.ygeno.2004.11.012 15718103

[B43] Linder P Owttrim GW Plant RNA helicases: linking aberrant and silencing RNA Trends Plant Sci 2009 14 344 352 10.1016/j.tplants.2009.03.007 19446493

[B44] Schultz J Milpetz F Bork P Ponting CP SMART, a simple modular architecture research tool: identification of signaling domains Proc Natl Acad Sci U S A 1998 95 5857 5864 10.1073/pnas.95.11.5857 9600884 PMC34487

[B45] Jermy AJ Willer M Davis E Wilkinson BM Stirling CJ The Brl domain in Sec63p is required for assembly of functional endoplasmic reticulum translocons J Biol Chem 2006 281 7899 7906 10.1074/jbc.M511402200 16368690

[B46] Ponting CP Proteins of the endoplasmic-reticulum-associated degradation pathway: domain detection and function prediction Biochem J 2000 351 Pt 2 527 535 11023840 PMC1221390

[B47] Doherty AJ Serpell LC Ponting CP The helix-hairpin-helix DNA-binding motif: a structural basis for non-sequence-specific recognition of DNA Nucleic Acids Res 1996 24 2488 2497 10.1093/nar/24.13.2488 8692686 PMC145986

[B48] Staub E Fiziev P Rosenthal A Hinzmann B Insights into the evolution of the nucleolus by an analysis of its protein domain repertoire Bioessays 2004 26 567 581 10.1002/bies.20032 15112237

[B49] Larocque JR Jasin M Mechanisms of recombination between diverged sequences in wild-type and BLM-deficient mouse and human cells Mol Cell Biol 2010 30 1887 1897 10.1128/MCB.01553-09 20154148 PMC2849462

[B50] Ngo HP Lydall D Survival and growth of yeast without telomere capping by Cdc13 in the absence of Sgs1, Exo1, and Rad9 PLoS Genet 2010 6 e1001072 10.1371/journal.pgen.1001072 20808892 PMC2924318

[B51] Ma E MacRae IJ Kirsch JF Doudna JA Autoinhibition of human dicer by its internal helicase domain J Mol Biol 2008 380 237 243 10.1016/j.jmb.2008.05.005 18508075 PMC2927216

[B52] Robb GB Rana TM RNA helicase A interacts with RISC in human cells and functions in RISC loading Mol Cell 2007 26 523 537 10.1016/j.molcel.2007.04.016 17531811

[B53] Zhou R Hotta I Denli AM Hong P Perrimon N Hannon GJ Comparative analysis of argonaute-dependent small RNA pathways in Drosophila Mol Cell 2008 32 592 599 10.1016/j.molcel.2008.10.018 19026789 PMC2615197

[B54] Tomari Y Du T Haley B Schwarz DS Bennett R Cook HA Koppetsch BS Theurkauf WE Zamore PD RISC assembly defects in the Drosophila RNAi mutant armitage Cell 2004 116 831 841 10.1016/S0092-8674(04)00218-1 15035985

[B55] Suzuki HI Yamagata K Sugimoto K Iwamoto T Kato S Miyazono K Modulation of microRNA processing by p53 Nature 2009 460 529 533 10.1038/nature08199 19626115

[B56] Jonassen I Collins JF Higgins DG Finding flexible patterns in unaligned protein sequences Protein Sci 1995 4 1587 1595 10.1002/pro.5560040817 8520485 PMC2143188

[B57] Faghiri Z Widmer G A comparison of the Giardia lamblia trophozoite and cyst transcriptome using microarrays BMC Microbiol 2011 11 91 10.1186/1471-2180-11-91 21542940 PMC3096902

[B58] Lujan HD Mowatt MR Byrd LG Nash TE Cholesterol starvation induces differentiation of the intestinal parasite Giardia lamblia Proc Natl Acad Sci U S A 1996 93 7628 7633 10.1073/pnas.93.15.7628 8755526 PMC38797

[B59] Birkeland SR Preheim SP Davids BJ Cipriano MJ Palm D Reiner DS Svard SG Gillin FD McArthur AG Transcriptome analyses of the Giardia lamblia life cycle Mol Biochem Parasitol 2010 174 62 65 10.1016/j.molbiopara.2010.05.010 20570699 PMC2972195

[B60] Morey JS Ryan JC Van Dolah FM Microarray validation: factors influencing correlation between oligonucleotide microarrays and real-time PCR Biol Proced Online 2006 8 175 193 10.1251/bpo126 17242735 PMC1779618

[B61] Etienne W Meyer MH Peppers J Meyer RA Jr Comparison of mRNA gene expression by RT-PCR and DNA microarray Biotechniques 2004 36 618 620 622, 624-616 15088380 10.2144/04364ST02

[B62] Morf L Spycher C Rehrauer H Fournier CA Morrison HG Hehl AB The transcriptional response to encystation stimuli in Giardia lamblia is restricted to a small set of genes Eukaryot Cell 2010 9 1566 1576 10.1128/EC.00100-10 20693303 PMC2950437

[B63] Chu CY Rana TM Translation repression in human cells by microRNA-induced gene silencing requires RCK/p54 PLoS Biol 2006 4 e210 10.1371/journal.pbio.0040210 16756390 PMC1475773

[B64] Fukuda T Yamagata K Fujiyama S Matsumoto T Koshida I Yoshimura K Mihara M Naitou M Endoh H Nakamura T DEAD-box RNA helicase subunits of the Drosha complex are required for processing of rRNA and a subset of microRNAs Nat Cell Biol 2007 9 604 611 10.1038/ncb1577 17435748

[B65] Naqvi AR Islam MN Choudhury NR Haq QM The fascinating world of RNA interference Int J Biol Sci 2009 5 97 117 19173032 10.7150/ijbs.5.97 PMC2631224

[B66] Ambrus AM Frolov MV The diverse roles of RNA helicases in RNAi Cell Cycle 2009 8 3500 3505 10.4161/cc.8.21.9887 19823018 PMC3016640

[B67] Morrison HG McArthur AG Gillin FD Aley SB Adam RD Olsen GJ Best AA Cande WZ Chen F Cipriano MJ Genomic minimalism in the early diverging intestinal parasite Giardia lamblia Science 2007 317 1921 1926 10.1126/science.1143837 17901334

[B68] Linder P Dead-box proteins: a family affair–active and passive players in RNP-remodeling Nucleic Acids Res 2006 34 4168 4180 10.1093/nar/gkl468 16936318 PMC1616962

[B69] Carranza PG Lujan HD New insights regarding the biology of Giardia lamblia Microbes Infect 2010 12 71 80 10.1016/j.micinf.2009.09.008 19772929

[B70] Lujan HD Mowatt MR Conrad JT Bowers B Nash TE Identification of a novel Giardia lamblia cyst wall protein with leucine-rich repeats. Implications for secretory granule formation and protein assembly into the cyst wall J Biol Chem 1995 270 29307 29313 10.1074/jbc.270.49.29307 7493963

[B71] Sun CH Palm D McArthur AG Svard SG Gillin FD A novel Myb-related protein involved in transcriptional activation of encystation genes in Giardia lamblia Mol Microbiol 2002 46 971 984 10.1046/j.1365-2958.2002.03233.x 12421304

[B72] Wang CH Su LH Sun CH A novel ARID/Bright-like protein involved in transcriptional activation of cyst wall protein 1 gene in Giardia lamblia J Biol Chem 2007 282 8905 8914 10.1074/jbc.M611170200 17244608

[B73] Sun CH Su LH Gillin FD Novel plant-GARP-like transcription factors in Giardia lamblia Mol Biochem Parasitol 2006 146 45 57 10.1016/j.molbiopara.2005.10.017 16310259

[B74] Pan YJ Cho CC Kao YY Sun CH A novel WRKY-like protein involved in transcriptional activation of cyst wall protein genes in Giardia lamblia J Biol Chem 2009 284 17975 17988 10.1074/jbc.M109.012047 19423705 PMC2709367

[B75] Sonda S Morf L Bottova I Baetschmann H Rehrauer H Caflisch A Hakimi MA Hehl AB Epigenetic mechanisms regulate stage differentiation in the minimized protozoan Giardia lamblia Mol Microbiol 2010 76 48 67 10.1111/j.1365-2958.2010.07062.x 20132448

[B76] Klappacher GW Lunyak VV Sykes DB Sawka-Verhelle D Sage J Brard G Ngo SD Gangadharan D Jacks T Kamps MP An induced Ets repressor complex regulates growth arrest during terminal macrophage differentiation Cell 2002 109 169 180 10.1016/S0092-8674(02)00714-6 12007404

[B77] Wilson BJ Bates GJ Nicol SM Gregory DJ Perkins ND Fuller-Pace FV The p68 and p72 DEAD box RNA helicases interact with HDAC1 and repress transcription in a promoter-specific manner BMC Mol Biol 2004 5 11 10.1186/1471-2199-5-11 15298701 PMC514542

[B78] Mooney SM Grande JP Salisbury JL Janknecht R Sumoylation of p68 and p72 RNA helicases affects protein stability and transactivation potential Biochemistry 2010 49 1 10 10.1021/bi901263m 19995069

[B79] Welker NC Maity TS Ye X Aruscavage PJ Krauchuk AA Liu Q Bass BL Dicer's helicase domain discriminates dsRNA termini to promote an altered reaction mode Mol Cell 2011 41 589 599 10.1016/j.molcel.2011.02.005 21362554 PMC3061311

[B80] Zhang YQ Chen DL Tian HF Zhang BH Wen JF Genome-wide computational identification of microRNAs and their targets in the deep-branching eukaryote Giardia lamblia Comput Biol Chem 2009 33 391 396 10.1016/j.compbiolchem.2009.07.013 19716768

[B81] Puntervoll P Linding R Gemund C Chabanis-Davidson S Mattingsdal M Cameron S Martin DM Ausiello G Brannetti B Costantini A ELM server: A new resource for investigating short functional sites in modular eukaryotic proteins Nucleic Acids Res 2003 31 3625 3630 10.1093/nar/gkg545 12824381 PMC168952

[B82] Sigrist CJ Cerutti L de Castro E Langendijk-Genevaux PS Bulliard V Bairoch A Hulo N PROSITE, a protein domain database for functional characterization and annotation Nucleic Acids Res 2010 38 D161 166 10.1093/nar/gkp885 19858104 PMC2808866

[B83] Gouy M Guindon S Gascuel O SeaView version 4: A multiplatform graphical user interface for sequence alignment and phylogenetic tree building Mol Biol Evol 2010 27 221 224 10.1093/molbev/msp259 19854763

[B84] Edgar RC MUSCLE: multiple sequence alignment with high accuracy and high throughput Nucleic Acids Res 2004 32 1792 1797 10.1093/nar/gkh340 15034147 PMC390337

[B85] Larkin MA Blackshields G Brown NP Chenna R McGettigan PA McWilliam H Valentin F Wallace IM Wilm A Lopez R Clustal W and Clustal X version 2.0 Bioinformatics 2007 23 2947 2948 10.1093/bioinformatics/btm404 17846036

[B86] Guindon S Gascuel O A simple, fast, and accurate algorithm to estimate large phylogenies by maximum likelihood Syst Biol 2003 52 696 704 10.1080/10635150390235520 14530136

[B87] Diamond LS Clark CG Cunnick CC YI-S, a casein-free medium for axenic cultivation of Entamoeba histolytica, related Entamoeba, Giardia intestinalis and Trichomonas vaginalis J Eukaryot Microbiol 1995 42 277 278 10.1111/j.1550-7408.1995.tb01579.x 7496385

[B88] Boucher SE Gillin FD Excystation of in vitro-derived Giardia lamblia cysts Infect Immun 1990 58 3516 3522 2228222 10.1128/iai.58.11.3516-3522.1990 PMC313691

[B89] Pfaffl MW Horgan GW Dempfle L Relative expression software tool (REST) for group-wise comparison and statistical analysis of relative expression results in real-time PCR Nucleic Acids Res 2002 30 e36 10.1093/nar/30.9.e36 11972351 PMC113859

