# Supplementary material for: Putative SF2 helicases of the early-branching eukaryote Giardia lamblia are involved in antigenic variation and parasite differentiation into cysts
Source: BMC Microbiol. 2012 Nov 28;12:284. doi: 10.1186/1471-2180-12-284 (PMC3566956; doi:10.1186/1471-2180-12-284)
Supplement: Additional file 1: Table S1 — Putative SF2 Helicases from Giardia lamblia. The table indicates the Family, the gene number from the Assemblage A isolate WB (the number that is given should be preceded by the prefix GL50803_), the current Supercontig or positions where it is located, the number of nucleotides in base pairs (bp) and molecular mass of the putative protein in kDa, for each putative helicase. (DOCX 18 kb) [file 1471-2180-12-284-S1.docx]

| **Additional file 1** | | | |
| --- | --- | --- | --- |
| **Putative SF2 Helicases from *Giardia lamblia****.* The table indicates the Family, the gene number from the Assemblage A isolate WB (the number that is given should be preceded by the prefix GL50803_), the current Supercontig or positions where it is located, the number of nucleotides in base pairs (bp) and molecular mass of the putative protein in kDa, for each putative helicase. | | | |
| **Family** | **GiardiaDB**  **gene number**  **(GL50803)** | **Supercontig [Location]** | **Size**  **bp/kDa** |
| **DEAD** | 2098 | CH991763 [864345 - 865637 (+)] | 1293/48.4 |
|  | 6283 | CH991768 [1188803 - 1190233 (+)] | 1431/53.9 |
|  | 9119 | CH991768 [203089 - 204441 (+)] | 1353/49.2 |
|  | 10255 | CH991762 [138060 - 139235 (+)] | 1176/43.2 |
|  | 13156 | CH991767 [109383 - 110669 (-)] | 1287/47.9 |
|  | 13220 | CH991814 [366102 - 368372 (+)] | 2271/84.4 |
|  | 13791 | CH991779 [1197337 - 1199214 (-)] | 1878/70.8 |
|  | 14098 | CH991767 [1214669 - 1216984 (+)] | 2316/86.1 |
|  | 14451 | CH991762 [365588 - 367252 (+)] | 1665/62.1 |
|  | 15048 | CH991768 [1297275 - 1299245 (-)] | 1971/73.3 |
|  | 15555 | CH991767 [1097784 - 1099976 (+)] | 2193/82.6 |
|  | 16042 | CH991767 [664969 - 666867 (-)] | 1899/70 |
|  | 16376 | CH991776 [20394 - 21944 (+)] | 1551/56.9 |
|  | 16806 | CH991779 [1405408 - 1407234 (+)] | 1827/67.2 |
|  | 16887 | CH991763 [909549 - 911192 (+)] | 1644/60.6 |
|  | 17239 | CH991782 [136732 - 138450 (+)] | 1719/63.4 |
|  | 17497 | CH991814 [428694 - 430940 (-)] | 2247/82.4 |
|  | 34684* | CH991776 [21991 - 23994 (+)] | 2004/74.4 |
|  | 90950 | CH991768 [1257745 - 1260447 (-)] | 2703/100.2 |
|  | 95898 | CH991771 [72852 - 74534 (-)] | 1683/62.6 |
|  | 96537 | CH991767 [1627234 - 1629084 (+)] | 1851/68.9 |
|  | 113655 | CH991782 [323813 - 325498 (+)] | 1686/62.9 |
| **DEAH** | 6616 | CH991767 [577536 - 579914 (+)] | 2379/87.8 |
|  | 13200* | CH991767 [234403 - 237803 (-)] | 1911/70.5 |
|  | 15930 | CH991763 [922033 - 924174 (+)] | 2142/79.3 |
|  | 17387 | CH991763 [700216 - 704652 (-)] | 4437/166.2 |
|  | 17539 | CH991768 [189279 - 191339 (+)] | 2061/76.6 |
|  | 92739 | CH991768 [688757 - 692506 (+)] | 3750/140.7 |
| **Ski2** | 9352 | CH991767 [517145 - 521929 (-)] | 4785/177.3 |
|  | 11384 | CH991769 [155212 - 157911 (-)] | 2700/98.9 |
|  | 17146 | CH991779 [91546 - 95631 (-)] | 4086/151.9 |
|  | 87022 | CH991779 [996103 - 1003368 (-)] | 7266/273.6 |
| **Swi2/Snf2** | 7890 | CH991817 [52487 - 56590 (+)] | 4104/154.1 |
|  | 8228 | CH991762 [56216 - 60046 (-)] | 3831/144.9 |
|  | 16143 | CH991782 [1063449 - 1069688 (+)] | 6240/232.7 |
|  | 16370 | CH991779 [957084 - 962663 (-)] | 5580/210.3 |
|  | 16512 | CH991779 [909034 - 911331 (-)] | 2298/88.2 |
|  | 87205 | CH991769 [491164 - 493956 (-)] | 2793/105.6 |
|  | 112978 | CH991763 [233669 - 241606 (-)] | 7938/300.6 |
| **RecQ** | 9145 | CH991767 [276975 - 279083 (-)] | 2109/78.6 |
|  | 9266 | CH991814 [240041 - 241957 (+)] | 1917/71.2 |
|  | 17438 | CH991779 [1203316 - 1205868 (-)] | 2553/95.2 |
| **Rad3** | 4328 | CH991771 [13514 - 16036 (+)] | 2523/94.1 |
|  | 5631 | CH991779 [102625 - 105804 (+)] | 3180/117.8 |
|  | 5910 | CH991762 [183507 - 185294 (-)] | 1788/67.7 |
|  | 92673 | CH991767 [1393611 - 1396037 (+)] | 2427/89.4 |

* The location is modified according with our analysis.
