# Supplementary material for: Putative SF2 helicases of the early-branching eukaryote Giardia lamblia are involved in antigenic variation and parasite differentiation into cysts
Source: BMC Microbiol. 2012 Nov 28;12:284. doi: 10.1186/1471-2180-12-284 (PMC3566956; doi:10.1186/1471-2180-12-284)
Supplement: Additional file 2: Table S2 — Average lengths (amino acid) of SF2 helicase families from Giardia lamblia. The table indicates the average length (in number of amino acids) of each SF2 helicase family. The incompletes sequences were not considered in the computation. [file 1471-2180-12-284-S2.docx]

| **Table S2** | | | | |
| --- | --- | --- | --- | --- |
| **Average lengths (amino acid) of SF2 helicase families from *Giardia lamblia***. The table indicates the average length (in number of amino acids) of each SF2 helicase family. The incompletes sequences were not considered in the computation. | | | | |
| **Family** | **Total** | **N-term** | **Helic. core domain** | **C-term** |
| **DEAD** | 600 | 86 | 367 | 147 |
| **DEAH** | 926 | 90 | 416 | 454 |
| **Ski2** | 1255 | 150 | 426 | 679 |
| **RecQ** | 730 | 94 | 359 | 277 |
| **Swi2/Snf2** | 1560 | 455 | 603 | 502 |
| **Rad3** | 825 | 38 | 708 | 79 |
