# Supplementary figures and images for: Putative SF2 helicases of the early-branching eukaryote Giardia lamblia are involved in antigenic variation and parasite differentiation into cysts
Source: BMC Microbiol. 2012 Nov 28;12:284. doi: 10.1186/1471-2180-12-284 (PMC3566956; doi:10.1186/1471-2180-12-284)

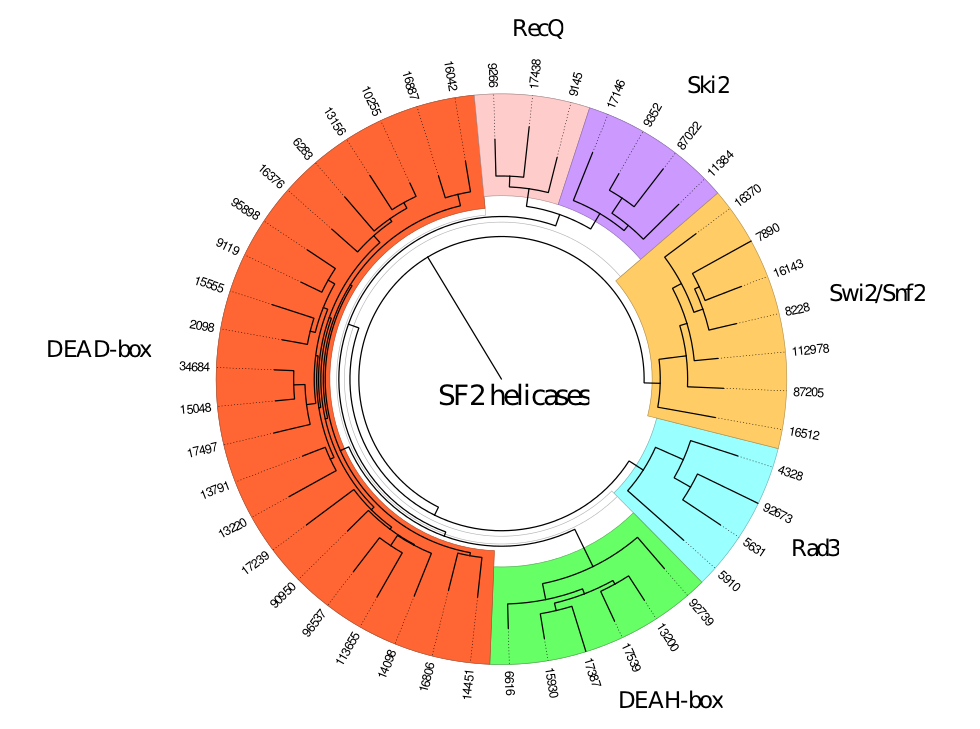

Supplement: Additional file 3: Figure S1 — Phylogenetic tree of the 46 putative SF2 helicase genes in Giardia lamblia. Phylogenetic tree derived from the alignment of the “Helicase Core Domain” amino acid sequences. Each helicase is named after its gene number, as in the GiardiaDB. The family groups are indicated as follows: DEAD-box (orange), DEAH-box (green), Ski2 (violet), RecQ (pink), Swi2/Snf2 (light orange) and Rad3 (light blue). [file 1471-2180-12-284-S3.png]

7890

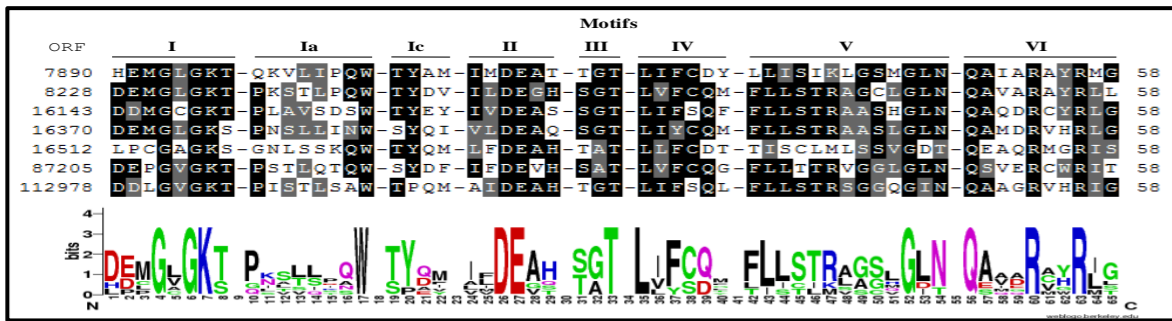

8228

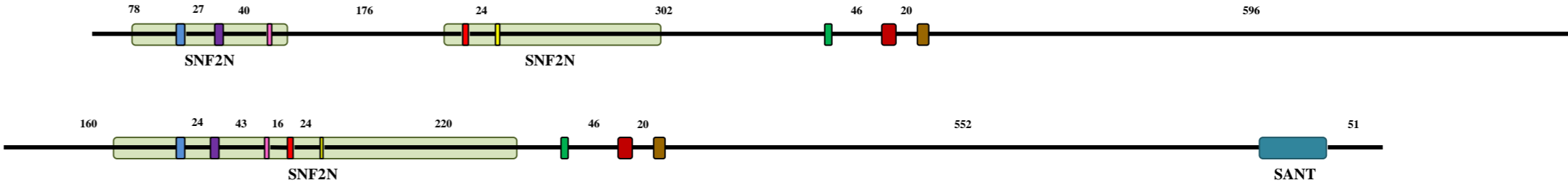

16143

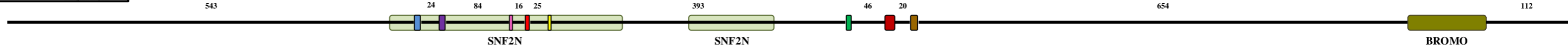

16370

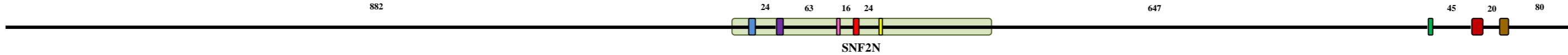

16512

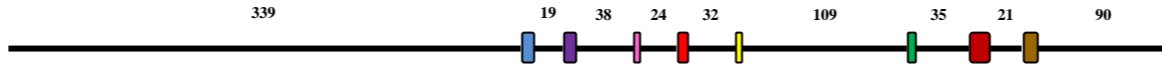

87205

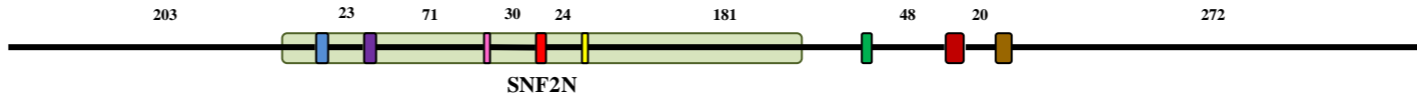

112978

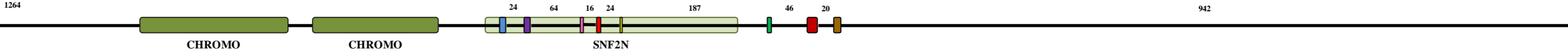

Supplement: Additional file 8: Figure S5 — Schematic diagram of the Swi2-Snf2 helicase family in G. lamblia. The SANT domain is represented in blue, the BROMO domain in brown, and the CHROMO domain in green. The SNF2N domains are represented in light grey, inside each one of them are the helicase motifs, when appropriate. The representation is to scale. Inset: sequence LOGO view of the consensus amino acids. The height of each amino acid represents the degree of conservation. Colors indicate properties of the amino acids, as follows: green (polar), blue (basic), red (acidic) and black (hydrophobic). [file 1471-2180-12-284-S8.pdf]

9145

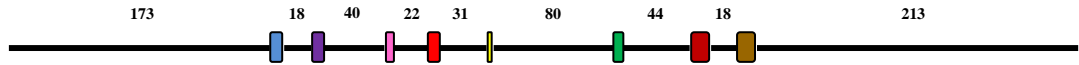

9266

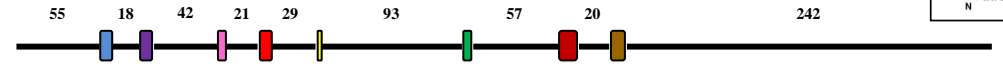

17438

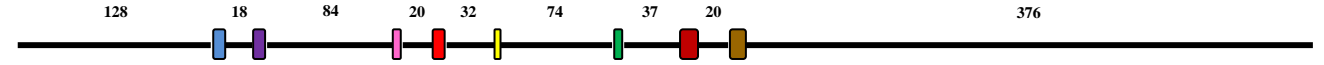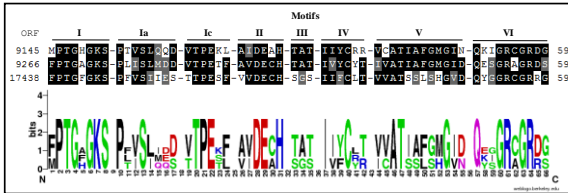

Supplement: Additional file 9: Figure S6 — Schematic diagram of the RecQ helicase family in G. lamblia. The representation is to scale. Inset: sequence LOGO view of the consensus amino acids. The height of each amino acid represents the degree of conservation. Colors mark properties of the amino acids as: green (polar); blue (basic); red (acidic) and black (hydrophobic). [file 1471-2180-12-284-S9.pdf]

4328

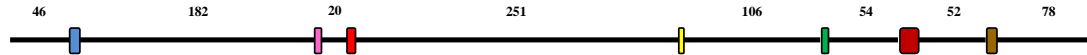

5631

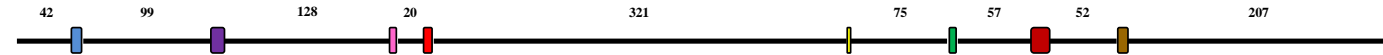

5910

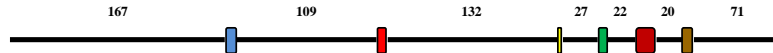

92763

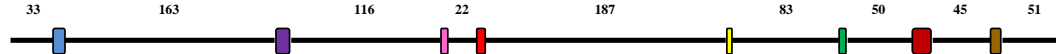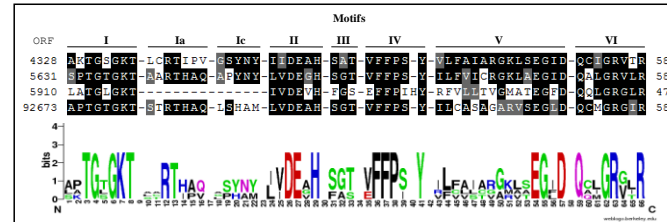

Supplement: Additional file 10: Figure S7 — Schematic diagram of the Rad3 helicase family in G. lamblia. The representation is to scale. Inset: sequence LOGO view of the consensus amino acids. The height of each amino acid represents the degree of conservation. Colors indicate properties of the amino acids, as follows: green (polar), blue (basic), red (acidic) and black (hydrophobic). [file 1471-2180-12-284-S10.pdf]

**Normal**

**Encyst**

49 -

37 -

26 -

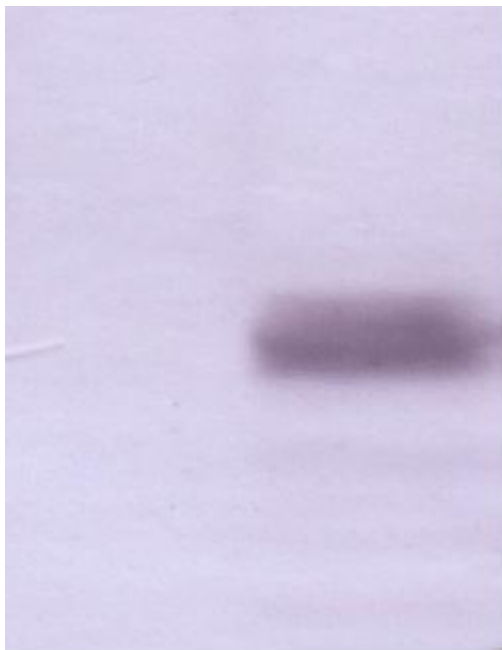

**W. blot**

**Normal**

**Encyst**

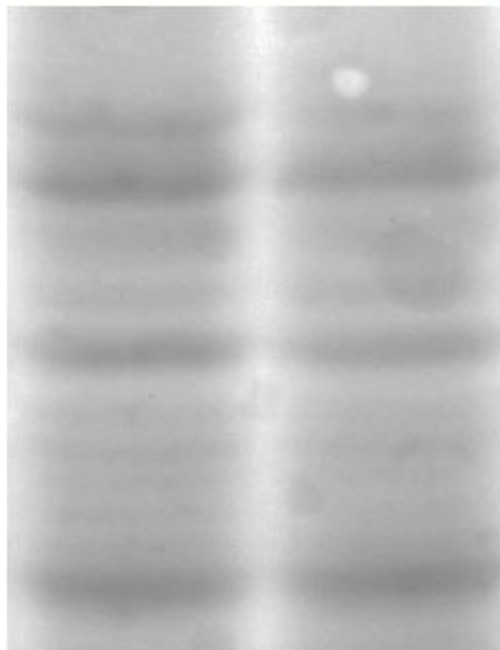

**Ponceau S**

Supplement: Additional file 11: Figure S8 — Western blot of trophozoites grown under proliferating conditions and after induction to encyst. Total protein extracts from trophozoites grown under normal proliferating conditions (Normal) or after 16hs induction in encystation medium (Encyst) were separated using a 10% SDS-polyacrylamide gel and transferred to a PVDF membrane. The membrane was incubated with a monoclonal antibody against CWP2. The iqual loading of the samples is shown in the figure at the right with a Ponceau S staining. The numbers indicate the molecular weight of protein standards in kDa. [file 1471-2180-12-284-S11.pdf]

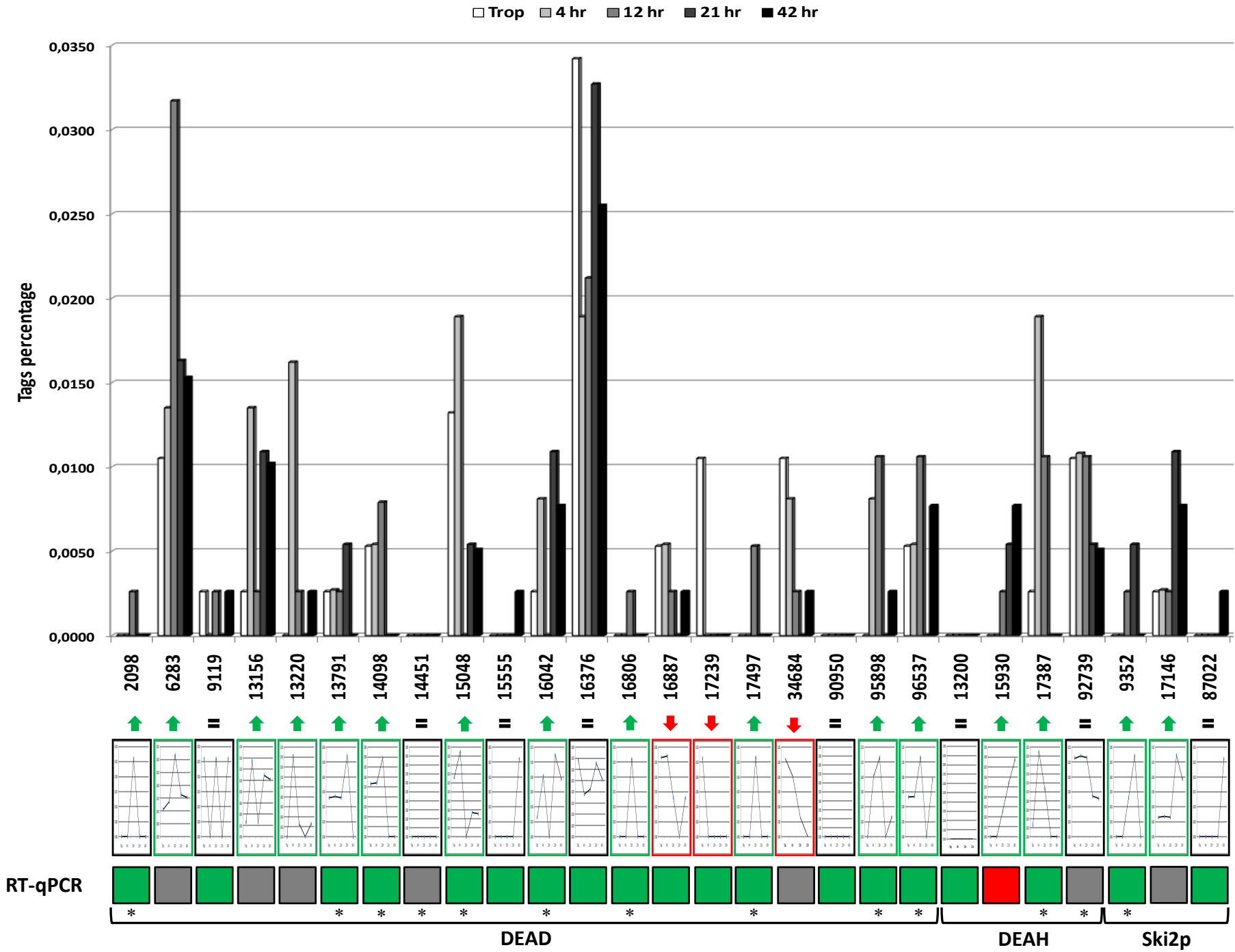

Supplement: Additional file 12: Figure S9 — SAGE (Serial Analysis of Gene Expression) data. The graph represents the sense tag percentage from Giardia trophozoites (white bar) and four different encystation times (4, 12, 21 and 42 hours; grayscale bars). Under each ORF it is indicated if these ORFs were up-regulated (green up arrow), down-regulated (red down arrow) or remained unmodified (equal sign). A line graph is also provided for a better identification of the expression pattern. The colored boxes represent our RT-qPCR results (with the same color code), divided into families. The asterisk under each box stands for a correlation between the SAGE and the RT-qPCR data. [file 1471-2180-12-284-S12.pdf]

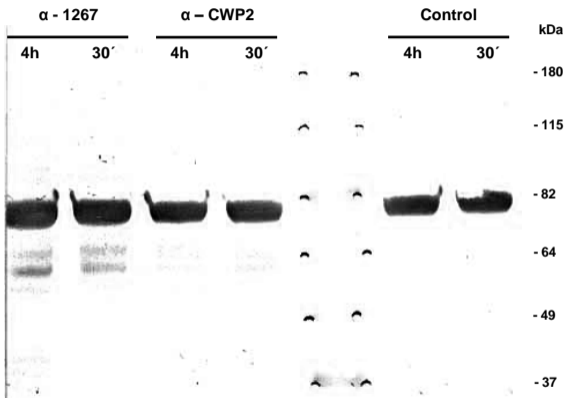

Supplement: Additional file 13: Figure S10 — Western blot during antigenic variation induction. Trophozoites were incubated for the indicated times with a 1:10.000 dilution of mAb 5C1directed against VSP-1267, mAb 7D2 against Cyst Wall Protein 2 or without antibody (Control). Total protein was electrophoresed, transferred to a PVDF membrane and incubated with a mAb against the VSP-1267. The molecular weights of standards are indicated in kDa. [file 1471-2180-12-284-S13.pdf]
