# Supplementary material for: Putative SF2 helicases of the early-branching eukaryote Giardia lamblia are involved in antigenic variation and parasite differentiation into cysts
Source: BMC Microbiol. 2012 Nov 28;12:284. doi: 10.1186/1471-2180-12-284 (PMC3566956; doi:10.1186/1471-2180-12-284)
Supplement: Additional file 4: Table S3 — Giardia lamblia SF2 helicases homologues in human and yeast. The table indicates each putative Giardia helicase with its Accession Number and ORF, the protein length in aminoacid, its putative helicase homologue form human with the identity and similarity percentage, and its putative helicase homologue from yeast with their known functions. [file 1471-2180-12-284-S4.docx]

| **Table S3** | | | | | | | | |  |
| --- | --- | --- | --- | --- | --- | --- | --- | --- | --- |
| *Giardia lamblia* SF2 helicases homologues in human and yeast | | | | | | | | |  |
| **Helicase** | | ***G. lamblia*** | **aa** | ***H. sapiens*** | **Percentage (%)** | | ***S.*** | **Yeast protein function** |  |
| **Super family** | **Family** | **Accession N°** (ORF) | **length** | (Synonyms) | **Identity** | **Similarity** | ***cerevisiae*** | **and other features** |  |
| **SF2** | **DEAD (22)** | **XM_001707073.1** (2098) | **430** | **DDX6**  (p54, HLR2) | **44** | **67** | **DHH1** | Stimulates mRNA decapping, coordinates distinct steps in mRNA function and decay, may have a role in mRNA export and translation |  |
|  |  | **XM_001709398.1** (6283) | **476** | **EIF4A3**  (DDX48, NUK34) | **27** | **47** | **FAL1** | Maturation of 18S rRNA, member of the eIF4A subfamily |  |
|  |  | **XM_001708809.1** (9119) | **450** | **DDX47**  (hqp0256 protein) | **41** | **58** | **RRP3** | rRNA processing; maturation of the 35S primary transcript of pre-rRNA and for cleavage leading to mature 18S rRNA |  |
|  |  | **XM_001709027.1** (10255) | **391** | **EIF4A3**  (DDX48, NUK34) | **47** | **69** | **FAL1** | Maturation of 18S rRNA, member of the eIF4A subfamily |  |
|  |  | **XM_001708445.1** (13156) | **428** | **EIF4A3**  (DDX48, NUK34) | **29** | **48** | **FAL1** | Idem |  |
|  |  | **XM_001707222.1** (13220) | **756** | **DDX24**  (CHL1-Like hel) | **24** | **43** | **MAK5** | Involved in biogenesis of large (60S) ribosomal subunit |  |
|  |  | **XM_001707624.1** (13791) | **625** | **DDX27**  (RHLP, Rrp3p) | **34** | **55** | **DRS1** | Ribosome assembly and function, including synthesis of60S ribosomal subunits; constituent of 66S pre-ribosomal particles |  |
|  |  | **XM_001707338.1** (14098) | **771** | **DDX31**  (FLJ13633/14578) | **29** | **44** | **DBP7** | Ribosomal biogenesis; essential for growth under anaerobic conditions |  |
|  |  | **XM_001704273.1** (14451) | **554** | **DDX52**  (ROK1) | **32** | **54** | **ROK1** | 18S rRNA synthesis |  |
|  |  | **XM_001705450.1** (15048) | **656** | **DDX3X**  (DBX, HLP2) | **34** | **50** | **DBP1** | Stability of the 40S ribosomal subunit scanning through 5' untranslated regions of mRNAs |  |
|  |  | **XM_001704330.1** (15555) | **730** | **DDX10**  (HRH-J8) | **36** | **53** | **HCA4** | 18S rRNA synthesis |  |
|  |  | **XM_001707463.1** (16042) | **632** | **DDX31**  (FLJ13633) | **32** | **50** | **DBP7** | Ribosomal biogenesis; essential for growth under anaerobic conditions |  |
|  |  | **XM_001705083.1** (16376) | **516** | **HLA-B**  (HLAC) | **36** | **57** | **SUB2** | Nuclear mRNA export; spliceosome assembly |  |
|  |  | **XM_001707761.1** (16806) | **608** | **DDX5**  (p68, Hlr1) | **28** | **45** | **DBP2** | Nonsense-mediated mRNA decay and rRNA processing |  |
|  |  | **XM_001707095.1** (16887) | **547** | **DDX18**  (MrDb) | **46** | **66** | **HAS1** | Highly enriched in nuclear pore complex fractions; constituent of 66S pre-ribosomal particles |  |
|  |  | **XM_001706250.1** (17239) | **572** | **DDX4**  (VASA) | **24** | **41** | **FAL1** | Maturation of 18S rRNA, member of the eIF4A subfamily |  |
|  |  | **XM_001707254.1** (17497) | **748** | **DDX43**  (HAGE) | **32** | **47** | **DBP2** | Nonsense-mediated mRNA decay and rRNA processing |  |
|  |  | **XM_001705084.1** (34684) | **449** | **DDX3Y**  (DBY) | **41** | **62** | **DED1** | Required for translation initiation of all yeast mRNAs |  |
|  |  | **XM_001705421.1** (90950) | **900** | **DDX54**  (DP97) | **38** | **56** | **DBP10** | Constituent of 66S pre-ribosomal particles; essential protein involved in ribosome biogenesis |  |
|  |  | **XM_001706855.1** (95898) | **560** | **DDX49**  (FLJ10432) | **40** | **55** | **DBP8** | Component of 90S preribosome complex involved in production of 18S rRNA and assembly of 40S small ribosomal subunit |  |
|  |  | **XM_001705931.1** (96537) | **616** | **DDX56**  (DDX21, DDX26) | **31** | **53** | **DBP9** | Biogenesis of the 60S ribosomal subunit |  |
|  |  | **XM_001704900.1** (113655) | **561** | **DDX51**  (MGC42193) | **23** | **39** | **DBP6** | Essential protein involved in ribosome biogenesis |  |
|  | **DEAH (6)** | **XM_001704665.1** (6616) | **792** | **DHX16**  (DBP2, PRP8) | **27** | **45** | **PRP2** | Activation of the spliceosome before the first transesterification step in RNA splicing |  |
|  |  | **XM_001708515.1** (13200) | **636** | **DHX8**  (HRH1, PRP22) | **29** | **48** | **PRP22** | Associates with lariat intermediates; mediates ATP-dependent mRNA release from the spliceosome and unwinds RNA duplexes |  |
|  |  | **XM_001707104.1** (15930) | **713** | **DHX35**  (FLJ22759) | **33** | **53** | **PRP43** | Functions in both RNA polymerase I and polymerase II transcript metabolism, involved in release of the lariat-intron from the spliceosome |  |
|  |  | **XM_001706117.1** (17387) | **1478** | **DHX37**  (KIAA1517) | **39** | **54** | **ECM16** | Specific to the U3 snoRNP, predominantly nucleolar in distribution, required for 18S rRNA synthesis |  |
|  |  | **XM_001708800.1** (17539) | **686** | **DHX15**  (DBP1, HRH2) | **29** | **47** | **PRP43** | Functions in both RNA polymerase I and polymerase II transcript metabolism, involved in release of the lariat-intron from the spliceosome |  |
|  |  | **XM_001708043.1** (92739) | **1249** | **DHX16**  (DBP2, PRP8) | **26** | **42** | **PRP2** | Activation of the spliceosome before the first transesterification step in RNA splicing |  |
|  | **SKI2 (4)** | **XM_001704958.1** (9352) | **1594** | **SNRNP200**  (BRR2, HELIC2) | **29** | **47** | **BRR2** | Disruption of U4/U6 base-pairing in native snRNPs to activate the spliceosome for catalysis |  |
|  |  | **XM_001706974.1** (11384) | **899** | **HELQ**  (HEL308; MGC20604) | **28** | **48** | **SLH1** | Translation inhibition of non-poly(A) mRNAs; required for repressing propagation of dsRNA viruses |  |
|  |  | **XM_001709468.1** (17146) | **1361** | **SKIV2L2**  (Dob1, Mtr4) | **43** | **63** | **MTR4** | Nuclear RNA processing and degredation both as a component of the TRAMP complex and in TRAMP ndependent processes |  |
|  |  | **XM_001704376.1** (87022) | **2421** | **ASCC3**  (RNAH, HELIC1) | **30** | **48** | **SLH1** | Translation inhibition of non-poly(A) mRNAs; required for repressing propagation of dsRNA viruses |  |
|  | **Swi2/Snf2 (7)** | **XM_001705007.1** (7890) | **1367** | **RAD54-like 2**  (ARIP4) | **25** | **41** | **RAD26** | Catalytic subunit of the SWI/SNF chromatin remodeling complex involved in transcriptional regulation |  |
|  |  | **XM_001708979.1** (8228) | **1276** | **SMARCA1**  (SNF2L) | **35** | **52** | **ISW2** | ATP-dependent DNA translocase involved in chromatin remodeling |  |
|  |  | **XM_001710193.1** (16143) | **2079** | **SMARCA4**  (BRG1) | **33** | **50** | **SNF2** | Catalytic subunit of the SWI/SNF chromatin remodeling complex involved in transcriptional regulation |  |
|  |  | **XM_001706082.1** (16370) | **1859** | **INO80**  (INOC1) | **37** | **55** | **INO80** | ATPase, subunit of a complex containing actin and several actin-related proteins that has chromatin remodeling activity |  |
|  |  | **XM_001706053.1** (16512) | **765** | **ERCC3**  (XPB; BTF2) | **37** | **58** | **SSL2** | Component of the RNA polymerase transcription factor TFIIH, has DNA-dependent ATPase/helicase activity and is required, with Rad3p, for unwinding promoter DNA |  |
|  |  | **XM_001706401.1** (87205) | **930** | **ERCC6**  (CSB; CKN2) | **33** | **50** | **RAD26** | Involved in transcription-coupled nucleotide excision repair of UV-induced DNA lesions |  |
|  |  | **XM_001705308.1** (112978) | **2645** | **CHD3**  (ZFH; Mi-2a) | **39** | **58** | **CHD1** | Nucleosome remodeling factor that functions in regulation of transcription elongation |  |
|  | **RecQ (3)** | **XM_001708540.1** (9145) | **702** | **BLM**  (RECQ2) | **28** | **43** | **SGS1** | Nucleolar DNA helicase of the RecQ family involved in genome integrity maintenance. Regulates chromosome synapsis and meiotic joint molecule/crossover formation |  |
|  |  | **XM_001708359.1** (9266) | **638** | **BLM**  (RECQ2) | **32** | **50** | **SGS1** | Idem |  |
|  |  | **XM_001707626.1** (17438) | **850** | **BLM**  (RECQ2) | **25** | **41** | **SGS1** | Idem |  |
|  | **Rad3 (4)** | **XM_001704074.1** (4328) | **840** | **ERCC2**  (EM9; TTD) | **27** | **47** | **RAD3** | 5' to 3' DNA helicase, involved in nucleotide excision repair and transcription |  |
|  |  | **XM_001709474.1** (5631) | **1059** | **RTEL1**  (NHL) | **35** | **51** | **RAD3** | Idem |  |
|  |  | **XM_001709054.1** (5910) | **595** | **NSSF*** |  |  | **IRC3** | Putative RNA helicase of the DEAH/D-box family |  |
|  |  | **XM_001706730.1** (92673) | **808** | **BRIP1**  (BACH1; FANCJ) | **31** | **48** | **CHL1** | Conserved nuclear protein required to establish sister-chromatid pairing during S-phase |  |
| **SF1** | **UPF1 (1)** | **XM_001707228.1** (13452) | **1304** | **UPF1**  (HUPF1, NORF1) | **39** | **52** | **NAM7** | Nonsense mediated mRNA decay; required for efficient translation termination at nonsense codons and targeting of NMD substrates to P-bodies; involved in telomere maintenance |  |
|  |  |  |  |  |  |  |  |  |  |
| **NSSF*:** *No Significant Similarity Found* | | | | | | | | | |
