# Supplementary material for: Putative SF2 helicases of the early-branching eukaryote Giardia lamblia are involved in antigenic variation and parasite differentiation into cysts
Source: BMC Microbiol. 2012 Nov 28;12:284. doi: 10.1186/1471-2180-12-284 (PMC3566956; doi:10.1186/1471-2180-12-284)
Supplement: Additional file 5: Figure S2 — Alignment of conserved DEAD-box helicase motifs. The sequences were aligned using the “Multiple Align Show” software at “The Sequence Manipulation Suite” (http://www.bioinformatics.org/sms/index.html). The residues conserved at 70% or more are highlighted in dark; other similar residues within each column are highlighted in grey. [file 1471-2180-12-284-S5.pdf]

Motifs

ORF	F	Q	I	Ia	Ic	II	III	IV	V	VI
13220	W	GYTKPRAIQ	AETGSGKT	IIAPTRELATQ	VGTPGRL	VIDESDKM	SAT	IIFVKT	LITTDVCARGLD	HIHRVGRAARVE
15555	W	SFTNMTPVQ	AETGSGKT	IVAPTQVLAQQ	VATPGRL	IIDEADQV	SAT	IVFFMT	LFTTDISARGLD	YTHRAGRTARCY
14098	F	GFKTMTPIQ	SRTGSGKT	ILLPTAELCVQ	IATPGRL	IFDEVDNL	TAI	VIFVAT	LITTDMCARGVD	YAHRAGRAARMN
90950	F	GYSTLTSIQ	SKTGSGKT	VIAPTRELCVQ	VCTPGRI	CFDESDRM	SAT	LVFVAT	LISTDVAARGID	YVHRVGRSARAG
17239	M	GYTSLRPIQ	SRTATGKT	VICPTRLLCEQ	VATPGIL	VLDEYDDL	SAT	IVFFLL	LFTTDLGARGLD	FIHRIGRAGRMG
2098	F	GFQQLTPVQ	AKNGTGKT	VLLHTRELAMQ	LATPGRL	VLDEADML	SAT	IVFVNS	LIATELFTRGID	YIHRAGRACRGD
16887	F	GHKNMTRIQ	AHTGSGKS	VLTPTRELALQ	IATPGRL	ILDEADML	SAT	IVFLSS	LLATNVAARGLD	YIHRAGRACRGD
95898	F	TITVPTDIQ	ARTGSGKT	VLTPTRELALQ	IATPGRL	VLDEADRL	TAT	IIFTDT	LVATDLASRGLD	YIHRVGRTCRAG
9119	F	GWLEPTAIQ	AETGSGKT	VLSPTRELAVQ	ICTPGRL	IIDEADKM	SAT	MVFATK	LLASDVAGRGID	YVHRAGRTARAG
13791	W	GWKFPTTVQ	AVTGSGKT	ILSPTRELAAQ	VATPGRL	VLDEGDKM	SAT	MVFCNS	LVCSDVASRGLD	HIHRVGRTGRAD
15048	F	KYFQPTPIQ	SQTGSGKT	IMSPTRELVQQ	VATPGRL	VFDECDRM	SAT	IVFTNF	LIGTDVAQRGLD	YTHRIGRTGRAG
34684	L	RYTQPTPIQ	SQTGSGKT	ILSPTRELTQQ	IATPGRL	VLDEADKM	SAT	LIFVET	LVATDVAQRGID	YIHRIGRTGRAG
16042	F	QVHNPTFIQ	AQTGSGKT	IVSPTKELAMQ	SGTPGRI	VLDECDQL	SAT	LVFAET	LVATDVAARGLN	YVHRVGRAGRMG
17497	F	KFSKPTCVQ	AETGSGKT	VFAPARELASQ	VTTPGIF	VLDECDAI	SAT	IVFVNK	LVATDAVARGVH	YVHRCGRTGRAG
6283	W	GFHMPTKTQ	APTGTGKT	IIAPTKILCRQ	IGTIHSL	IIDEADYF	SAT	IIFCKT	LITTDGLARGID	YTHRIGRGGRFG
13156	F	GFEIPSPVQ	AQTGSGKT	IISPTKELSNQ	CVTPKRL	ILDECDKL	SAT	VIFCNS	LISTDLLARGFD	YMHRAGRCGRFG
10255	F	GYKIPSAIQ	AQSGTGKT	ILSPTRELALQ	VATPGRL	VLDEADEM	SAT	VIFCNS	LIATNIIARGID	YLHRIGRSGRFG
16376	F	GFESPSDVQ	AKSGKGKT	VLCNTHELAMQ	VGTIGRV	VLDEFDAL	TAT	VIFARD	LVSTDIFQRGVD	YLHRSGRAGRFE
14451	F	GWEVPSPVQ	APTGSGKS	VLVPTRELADQ	VATPLVL	VLDEVDCL	SAS	LVFAST	LVCTDVLARGLD	YIHRIGRAGRQT
16806	W	EYTQLTPVQ	APTGSGKT	VLTPTRELAMQ	VGVPGKV	VLDELDEL	TAT	IIFLAS	LITTSGGGRGLD	YVHQIGRTARGM
96537	W	LYEAL-PIQ	SFTGSGKT	IVVPTRELCRQ	ISTPSLV	VCDEADLI	SAT	VIFVNS	-------FRGLD	YIHRIGRTARGA
113655	W	GYTDLLPIQ	SATGSGKT	FVVPTTHLLTQ	CGTPGIV	VIDEADHI	SAT	IVFVNS	VLATDNFARGLD	LTHRVGRCARGL
