# Supplementary material for: Putative SF2 helicases of the early-branching eukaryote Giardia lamblia are involved in antigenic variation and parasite differentiation into cysts
Source: BMC Microbiol. 2012 Nov 28;12:284. doi: 10.1186/1471-2180-12-284 (PMC3566956; doi:10.1186/1471-2180-12-284)
Supplement: Additional file 6: Figure S3 — Alignment of conserved DEAH-box helicase motifs. The sequences were aligned using the “Multiple Align Show” as before. The residues conserved at 70% or more are highlighted in dark; other similar residues within each column are highlighted in grey. [file 1471-2180-12-284-S6.pdf]

Motifs

ORF	I	Ia	Ib	II	III	IV	V	VI
17387	GSTGSGKS	VTQPRRIAAIG	FVTEGVLLKM	ILDEAHER	SAT	LIFLSG	TNVAEASITIPN	ADQRAGRAGRTA
15930	GSTGSGKS	VTQPRRVAAIS	YATDGVVIRE	IVDEAHER	SAT	LVFMPG	TNIAETSITVPN	ILQRSGRAGRLM
6616	GATGSGKS	VSEPRRVAAIS	YQTDGVTLQY	ILDEIHER	SAT	LVFLSG	TNVAETSVTIPG	HMQRRGRVGRTQ
17539	AETGSGKS	VVQPRRIAAVS	YVTEGILLNW	ILDEVHER	SAT	LVFLPG	TNIAETSLTIPG	ADQRKGRAGREG
92739	GPTGCGKS	LVQPRRLPTER	IATAGAAFNR	ILDEIHEE	SAT	IVFLPG	TNLCETSLTLPT	MIQRKGRVGRVR
13200	--------	VTQPRRVSAIS	YATEGILLRM	LIDEVHEK	SAT	LVFLPG	TNVAETSITIPD	AVQRKGRAGRVQ
