# Supplementary material for: Putative SF2 helicases of the early-branching eukaryote Giardia lamblia are involved in antigenic variation and parasite differentiation into cysts
Source: BMC Microbiol. 2012 Nov 28;12:284. doi: 10.1186/1471-2180-12-284 (PMC3566956; doi:10.1186/1471-2180-12-284)
Supplement: Additional file 7: Figure S4 — Alignment of conserved Ski2 helicase motifs. The sequences were aligned using the “Multiple Align Show” as before. The residues conserved at 70% or more are highlighted in dark; other similar residues within each column are highlighted in grey. [file 1471-2180-12-284-S7.pdf]

Motifs

ORF	I		Ia		Ic		II		III		IV		V		VI
9352	APTGAGKT	-	YVAPLKALVHE	-	ATPEKL	-	VFDELH	-	SA-T	-	LLFTHTR	-	TATLAWGVNMPA	-	QMAGRAGR
11384	APTSAGKS	-	IIAVPFVALAD	-	CTFEKA	-	VFDEIH	-	SA-T	-	IIFVTTK	-	TTTISAGVNLPA	-	QMIGRAGR
17146	AHTSAGKT	-	YTTPIKALSNQ	-	MTTEIL	-	IFDEVH	-	SA-T	-	IVFAFGK	-	TETFAMGLNLPA	-	QMAGRAGR
87022_R1	APTGAGKT	-	YLTPMKALASE	-	CTPEKW	-	IIDEIH	-	SA-T	-	LVFVHSR	-	TATLAWGVNMPC	-	QIFGRAGR
87022_R2	CPTGSGKT	-	YIAPMKALIRE	-	ATPEKF	-	IFDELH	-	SANT	-	LIFVASR	-	TSTLAWGLNLPA	-	QMAGRAGR
